# Constructing local cell-specific networks from single-cell data

**DOI:** 10.1073/pnas.2113178118

**Published:** 2021-12-13

**Authors:** Xuran Wang, David Choi, Kathryn Roeder

**Affiliations:** ^a^Department of Statistics and Data Science, Carnegie Mellon University, Pittsburgh, PA 15213;; ^b^Heinz College, Carnegie Mellon University, Pittsburgh, PA 15213;; ^c^Computational Biology Department, Carnegie Mellon University, Pittsburgh, PA 15213

**Keywords:** coexpression network, differential network genes, differential expression, single-cell RNA-seq, brain cells

## Abstract

Gene coexpression networks yield critical insights into biological processes, and single-cell RNA sequencing provides an opportunity to target inquiries at the cellular level. However, due to the sparsity and heterogeneity of transcript counts, it is challenging to construct accurate gene networks. We develop an approach, locCSN, that estimates cell-specific networks (CSNs) for each cell, preserving information about cellular heterogeneity that is lost with other approaches. LocCSN is based on a nonparametric investigation of the joint distribution of gene expression; hence it can readily detect nonlinear correlations, and it is more robust to distributional challenges. Although individual CSNs are estimated with considerable noise, average CSNs provide stable estimates of networks, which reveal gene communities better than traditional measures. Additionally, we propose downstream analysis methods using CSNs to utilize more fully the information contained within them. Repeated estimates of gene networks facilitate testing for differences in network structure between cell groups. Notably, with this approach, we can identify differential network genes, which typically do not differ in gene expression, but do differ in terms of the coexpression networks. These genes might help explain the etiology of disease. Finally, to further our understanding of autism spectrum disorder, we examine the evolution of gene networks in fetal brain cells and compare the CSNs of cells sampled from case and control subjects to reveal intriguing patterns in gene coexpression.

## Significance

Understanding gene regulatory networks is a topic of great interest because it can provide insights into cellular development, and identify factors that differ between normal and abnormal cells and phenotypes. Single-cell RNA sequencing provides a unique opportunity to gain understanding at the cellular level, but the technical features of the data create severe challenges when constructing gene networks. We develop a method that successfully skirts these challenges to estimate a cell-specific network for each single cell and cell type. Application of our algorithm to two brain cell samples furthers our understanding of autism spectrum disorder by examining the evolution of gene networks in fetal brain cells and comparing the networks of cells sampled from case and control subjects.

Single-cell RNA sequencing (scRNA-seq) provides a high throughput profile of RNA expression for individual cells that reveals the heterogeneity across cell populations. Recent advances in computational methods enable cell type classification, novel cell type identification (1), and trajectory alignment (2); however, among these methods, less attention has been devoted to gene–gene association and transcriptional networks, which can shed light on many vital biological processes. Gene expression is known to be tightly regulated by networks of transcription factors, and understanding these networks at a cellular level can identify differences in the inner workings of cells in normal and diseased tissues (3–6).

Thus far, efforts to estimate coexpression networks from scRNA-seq have been limited in their success (7, 8) for several reasons, including technical challenges such as a sparsity of nonzero counts and a high level of noise (9), nonlinear relationships that are not easily captured by traditional measures, and heterogeneity in coexpression patterns across cells. Attempts have been made to circumvent these challenges (4, 5, 10–12). Many approaches aim to estimate a single coexpression network across all cell types, but, to gain access to the subtle differences in gene coexpression at the cellular level, we require estimates of gene networks evaluated for each cell type. Moreover, even if the aim is to estimate a cell-type-specific network, traditional methods estimate a single coexpression relationship across the entire sample of cells of that type. With such an approach, heterogeneity of coexpression across individual cells is erased, but this knowledge can provide valuable insights for downstream analysis.

**Fig. 1.**
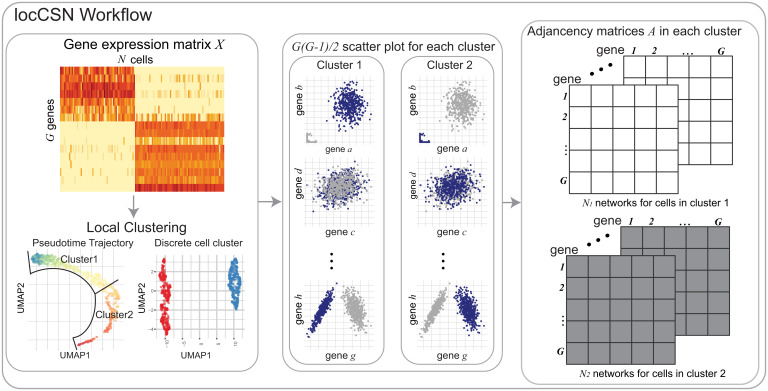
Workflow of locCSN. Starting with the gene expression matrix, we cluster homogeneous cells. For each cell in a cluster, we examine the coexpression of each pair of genes to determine whether the joint expression is unusually dense in a neighborhood of the cell, relative to expectation assuming the genes are independent; if so, the genes are connected in that cell’s network. For each cell, we then construct the gene–gene network, based on the results of the local independence test for each pair of genes.

To reveal coexpression networks within individual cells, we aim to construct cell-specific networks (CSNs) from scRNA-seq data. This idea was first proposed by Dai et al. (13) and Li et al. (14) wherein they determine gene–gene connectivity at a single-cell level. The construction of CSNs is based on the following assessment of local statistical dependency of a pair of genes: For an individual cell, if the coexpression of the pair of genes is unusually high, relative to their distribution assuming the genes to be independent, then the original CSN (oCSN) algorithm infers a gene–gene relationship (Fig. 1). Notably, the oCSN algorithm ignores the confounding effect of cell clusters, which can produce connections in the network between genes that are correlated globally, but are not correlated within a cell type (i.e., Simpson’s paradox). More importantly, for each cell, oCSN only records the degree sequence of its CSN, and this gene-by-cell matrix is used as input for clustering and low-dimensional embedding.

Relying on the CSN principle, a full gene–gene network is potentially available for each cell (although with high noise), which can be envisioned as a graph, with genes as nodes and edges depicting gene–gene dependencies. Building on this concept, we develop an alternative analysis scheme that utilizes a more powerful method, locCSN (https://github.com/xuranw/locCSN), and expands the utility of the CSN concept for gaining insights into cell biology. In particular, we retain the networks, rather than just their degree sequences, so that differences between cell groups and developmental changes can be examined at the level of the gene pair. First, to mitigate noise, individual networks may be averaged across cells within a category or developmental period, providing a nonparametric estimator of gene coexpression that performs well for the sparse counts generally observed for scRNA-seq data. This facilitates contrasts between gene networks of related cell types and allows for estimation of evolving gene communities in developmental trajectories. Second, having a sample of individual networks provides a valuable measure of the variability across cells that can be leveraged in powerful tests of network differences between cell groups. For example, comparisons can be made between cells obtained from case and control subjects, or cells sampled from different spatial or temporal regimes. Finally, the CSN approach facilitates follow-up analysis to discover the key genes driving the differences between networks. With this approach, we can identify “differential network (DN) genes,” which typically do not differ in gene expression, but do differ in terms of the coexpression network. These genes could play a key role in network regulation and hence differences that might help explain the etiology of disease.

We illustrate the value of locCSN by applying it to several scRNA-seq datasets, including developing fetal brains (15), and case and control samples from autism spectrum disorder (ASD) subjects (16). The locCSN extracts cell-level network information from these data which preserves cell-level network heterogeneity and highlights network differences between case and control samples, shedding light on important functional differences.

## Results

Our results are summarized as follows. First, we describe the locCSN algorithm. Second, simulated and real data examples are used to illustrate differences between locCSN and oCSN. Then, we apply time-varying community detection to CSNs taken from two overlapping developmental trajectories in the developing cortex atlas dataset, and identify differences in their community evolution. Finally, we show that CSNs enable better separation of case and control populations in the ASD brain dataset, by identifying genes that have differing coexpression (but similar expression levels) between the two groups.

### CSN Construction

The algorithm for construction of CSN as originally published (13) (oCSN) is to estimate, for each cell, a connection strength between each pair of genes, resulting in a gene–gene network for every cell. This connection strength is based on a local independence test that is applied to each gene pair and each cell. The test is nonparametric and does not impose assumptions of (nor distinguish notions of) linearity or monotonicity, and consists of comparing the joint density in a local region to the product of the marginals. To motivate this test, Dai et al. (13) give examples where the test succeeds in separating a mixture of cells, where genes *x* and *y* follow a dependent relationship in some cells but are independent in others (*SI Appendix*, Fig. S1*A*). However, the test requires a choice of resolution (i.e., a bandwidth or window size), which greatly affects performance. Dai et al. (13) proposed a fixed quantile range for the window size, but we find that this approach gives counterintuitive results and low power when the joint distribution of genes *x* and *y* is a simple correlated normal (*SI Appendix*, section 1 and Figs. S1 and S2). To improve performance, we propose an algorithm for CSN construction (locCSN) that allows for the window size of the local independence test to vary cell by cell.

While Dai et al. (13) use all expressions of all cell types to construct each CSN, we recommend that each CSN be constructed using only the cells of a common type, so that the independence test is conditional on cell type. Otherwise, false edges may be detected due to Simpson’s paradox: Given a pair of marker genes that display independent expression within the marked cell type, CSN construction on all cell types together will likely infer an edge indicating nonindependence of the genes due to the differential expression across types. Therefore, we recommend clustering the cells into distinct cell types, and then applying locCSN separately to each cluster, as illustrated for a single cluster in Fig. 1. Similarly, if cells are developing along a smooth trajectory, then the cells can be windowed according to pseudotime (2), and each CSN can be computed using the cells within its pseudotime bin.

For each cell, locCSN produces a test statistic for each pair of genes, *z_xy_*, to evaluate pairwise gene–gene independence in the neighborhood of that cell’s expression. By thresholding these tests at a given significance level (such as α=0.05 or 0.01), we obtain a zero–one adjacency matrix for each individual cell. Averaging the adjacency matrices gives an aggregate measure for the intensity of coexpression, which is positive by design and equals, for each gene pair, the fraction of cells for which the independence test is rejected.

Implementation of locCSN requires two key choices: an initialization of the window width for each local test and the thresholding parameter *α* to derive the zero–one adjacency matrix from the matrix of local test statistics. We simulate coexpression networks using ESCO (17) to evaluate performance of network estimation for various choices of these tuning parameters. We observe that the SD approach for window width recommended by locCSN performs markedly better than the quantile approach utilized by oCSN. The performance is robust to choice of starting value for the SD algorithm (*SI Appendix*, section2 and Figs. S3–S7). Our results also show that there is a range of threshold *α* for which performance is stable, and the optimal choice varies somewhat depending on the strength of the true correlations. Nevertheless, for both moderate and strong correlations, performance is good between 0.05 and 0.01, after which the accuracy drops precipitously (*SI Appendix*, section2 and Figs. S5 and S6).

### Illustrative Examples

To illustrate the importance of local calculations, we compare locCSN and oCSN for synthetic data from ESCO (17). The cells are sampled from two populations, one for which a set of genes exhibit pairwise correlation and another for which the genes are independent (*SI Appendix*, section 2 and Fig. S8 *A*–*C*). When calculated for this set of genes, ideally, the test statistics for each cell will distinguish the pattern of correlation present in one population (group2) not in the other (group1) (*SI Appendix*, Fig. S8*C*). The desired pattern is achieved by locCSN, but oCSN produces false signals of correlation for many cells in group1 (*SI Appendix*, Fig. S8 *D* and *E*). Mixing the two populations leads to false positives in group1, suggesting that the false positives result from Simpson’s paradox. Likewise, simulations show that BigSCale (12), a powerful alternative approach for coexpression estimation, yields many false connections not detected by locCSN (*SI Appendix*, Fig. S8*F*).

**Fig. 2.**
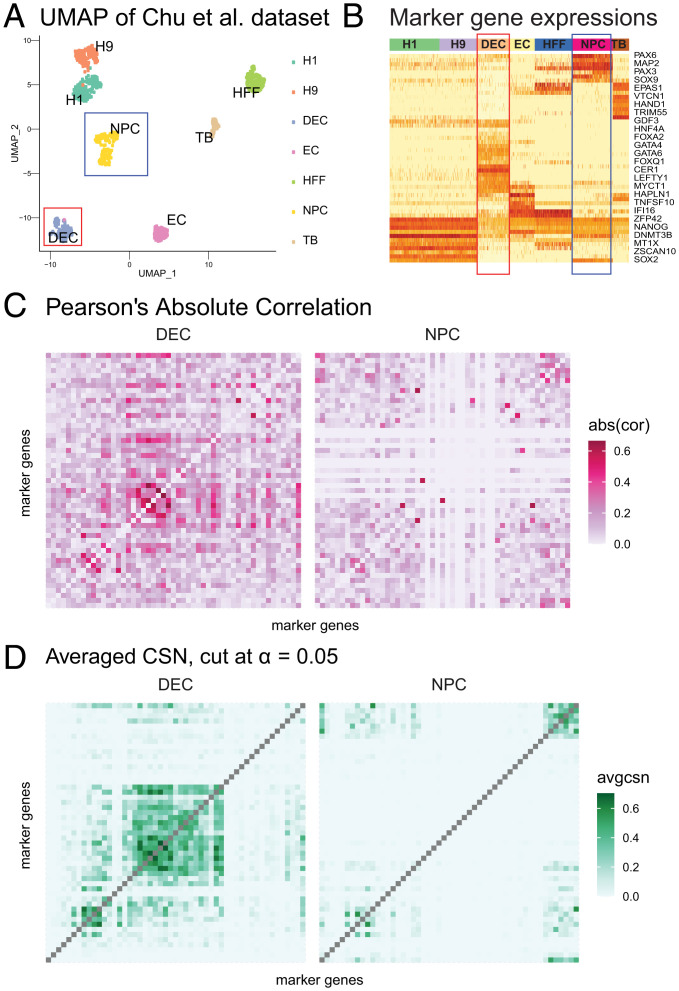
Estimated networks for Chu et al. (18) cells. (*A*) UMAP of Chu et al. cells, colored by cell types. The red and blue boxes indicate selected cell type: DEC and NPC. (*B*) Heatmap of gene expression for 57 developmental genes for seven cell types. High to low expression corresponds to red to light yellow in the heatmap. Seven cell types are color coded by the band on the top of the heatmap. The red and blue boxes indicate DEC and NPC cell types. (*C*) Heatmaps of absolute Pearson’s correlation for DEC and NPC cell types, calculated independently within cell types. The order of genes is the same as *B*. (*D*) Heatmaps of averaged locCSN for DEC and NPC cell types, thresholded at Gaussian distribution α=0.05 quantile. The order of genes is the same as *B*.

To demonstrate and evaluate the performance of CSN test statistics on real data, we use the Chu et al. (18) dataset. The Chu et al. dataset includes seven cell types (Fig. 2*A*). Marker genes corresponding to developmental lineage are provided by the authors, and a heatmap of gene expression reveals that a subset of genes mark cell types DEC and NPC fairly well (Fig. 2*B*). We analyze these two cell types, which contain 138 cells and 173 cells, respectively. The absolute Pearson’s correlation for lineage marker genes, computed within DEC and NPC cell types, does not show a clear pattern; specifically, the correlation does not delineate the expected block structure for marker genes for DEC and NPC cells (Fig. 2*C*). By contrast, averaged locCSNs, thresholded at α=0.05, preserve the gene block structures and emphasize the differences between cell types (Fig. 2*D*). We conclude that CSN works well on distinct cell types. The averaged CSN preserves gene blocks, distinguishes between cell types, and depicts a clearer coexpression pattern than Pearson’s correlation. By contrast, the averaged CSNs calculated from oCSN ignore coexpression between genes, especially the dense block for NPC at the upper right corner (*SI Appendix*, Fig. S9). Finally, we show that our results are robust to a range of tuning parameters (*SI Appendix*, Fig. S9 *B* and *C*).

The observed performance advantage of locCSN relative to Pearson’s is supported by simulations (*SI Appendix*, Fig. S7 and Table S1). These results are notable because the simulation generates a linear relationship between correlated genes. Thus, even when the correlation between genes follows the assumed parametric form, locCSN performs better because it adapts to the sparsity expected in scRNA-seq data.

### CSN Analysis of Developmental Trajectories

**Fig. 3.**
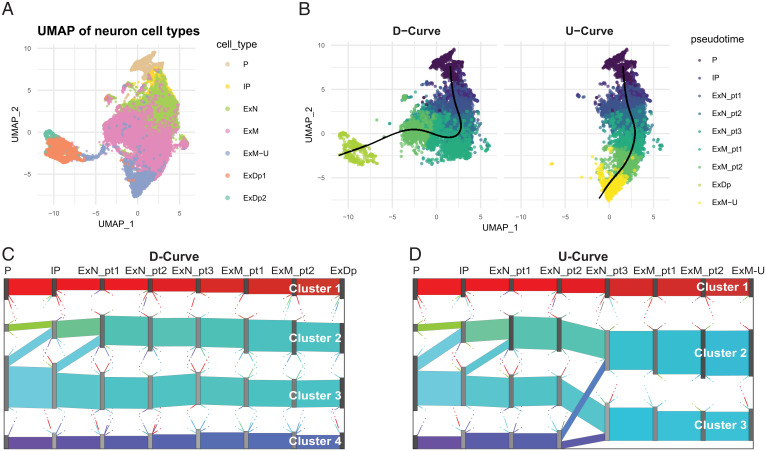
Development of networks in human fetal brain cells. (*A*) UMAP of human fetal brain single-cell expression from seven cell types involved in development of excitatory neuron cells, (*B*) with developmental trajectories superimposed. The UMAP plots indicate metacells, whose coordinates are the average UMAP cellular locations. The two principal curves are generated by Slingshot (19). Colors are determined by the metacell’s pseudotime and cell types for the two curves, which are calculated as the average pseudotime over all cells in the metacell. *Left* and *Right* show the metacells’ assignment, based on pseudotime, to D curve and U curve, respectively. (*C* and *D*) Sankey plots of averaged CSN for eight bins in D curve and U curve. Gene flows are shown as colored bands connecting two adjacent pseudotime bins for (*C*) D curve and (*D*) U curve. The color of each band is the mixture of colors assigned to its constituent genes, so that flows with similar gene compositions are assigned similar colors.

To illustrate the application of CSN to developing cells, we feature the developmental trajectory of human excitatory neuron cells in the Developing Cortex Atlas dataset (15), which includes Radial Gila and Progenitors (P), Intermediate Progenitors (IP), Maturing Excitatory (ExN), Migrating Excitatory (ExM), Excitatory Upper-layer enriched (ExM-U), and Excitatory Deep layer (ExDp), with cell type labels determined by the authors (*SI Appendix*, section 3 and Table S2). Using Slingshot (19), we estimate the developmental path consists of two trajectories, one ending in upper-layer (U curve) and the other in deep-layer (D curve) excitatory neurons (Fig. 3 *A* and *B*).

To circumvent challenges due to sparse counts, which are prevalent in these data, we pool similar cells within cell type and form metacells (20), each containing ∼20 cells. Each metacell’s expression, pseudotime, and Uniform Manifold Approximation and Projection (UMAP) coordinate is computed as the average over pooled cells. Metacells are assigned to trajectories based on proximity, using Slingshot (19). At the early stages of development, the curves are nearly overlapping, and metacells are not differentiated by curve, but, as the cells develop, metacells can be assigned to a distinct curve (*SI Appendix*, Tables S2 and S3). Both curves start at the P cell class with full overlap, but, moving along ExN and ExM cell classes, there is progressively less overlap between the two curves until they bifurcate, with D curve and U curve culminating in purely ExDp and ExM-U metacells, respectively. Next, within each cell type, we generate eight bins based on the metacell pseudotime values, each consisting of ∼800 metacells containing fairly homogeneous cells.

For each metacell, we use locCSN to compute gene networks. For illustration, we focus on a restricted gene list. We choose ASD risk genes because the development of excitatory neurons is of interest in ASD research (15, 21, 22). Specifically, CSNs were computed for 444 genes chosen by intersecting the expressed genes in the metacells with a list of 992 ASD-associated genes gleaned from the Simons Foundation Autism Research Initiative (SFARI) database (classes S, 1, 2, and 3) (23). Our objective is to map the formation of gene clusters over developmental epochs, as cells develop into upper- or deep-layer excitatory neurons. We apply PisCES (11) to the average CSNs per bin, to find time-varying gene community structure in the D-curve and U-curve trajectories, and use Sankey plots to visualize the evolution of gene communities (Fig. 3 *C* and *D*). As expected, the gene communities are nearly identical for the two curves for the first four pseudotime bins (Dataset S1), which share a large fraction of metacells, but the results progressively differentiate as the overlap in metacells diminishes. Most notably, the gene community associated with cluster 4 (purple) for U curve splits and merges into the other gene communities at ExN_pt3, while it persists for D curve. For both curves, cluster 1 (red) contains genes that are more highly connected than the other loosely connected gene communities (*SI Appendix*, Fig. S10). For all of the remaining identified gene clusters, the correlation is extremely weak in the early stages and becomes more apparent in the final developmental bin.

We study the membership in gene clusters when they are relatively stable without major splitting and merging from ExN_pt3 an onward. We refer to gene clusters in D curve as clusters 1 to 4 and, in U curve, as clusters 1 to 3, where labels run from top to bottom of the display. The numbers of genes in each stable cluster and the overlap between clusters from two curves are shown in *SI Appendix*, Table S4 (Dataset S1). For example, most of the genes from cluster 1 (dense cluster) for D curve are included in the corresponding dense cluster, that is, cluster 1 for U curve. While the cluster membership is fairly stable along each curve, the strength of correlation changes over time (*SI Appendix*, Fig. S10).

We also consider the averaged metacell gene expression across pseudotime for each gene cluster (*SI Appendix*, Fig. S11). Gene expression is relatively stable across cellular development. The dense gene cluster (cluster 1) has high expressions, while the loose gene clusters have relatively low expression. Yet, even though all genes outside of the dense cluster have lower expression levels, using locCSN, combined with PisCES, we are able to detect subtle correlation, and partition genes into gene communities. It is worth noting that none of these communities are identified by WGCNA (24) (*SI Appendix*, Fig. S12), which relies on Pearson’s correlation, but could also be implemented using average CSN matrices for potentially better performance.

To understand the function of gene communities, we check Gene Ontology (GO) terms (25) (*SI Appendix*, section 4) for the seven gene communities (*SI Appendix*, Fig. S13 and Dataset S1). For both curves, GO term treemaps for the dense gene community (cluster 1) include metabolic processes, organelle organization, and the process of mitosis, which are critical during the fetal stage. For D curve, cluster 2 is enriched for chromatin organization, which is critical for gene expression regulation, while, for U curve, cluster 2 is enriched for organelle and cellular component organization. The most loosely structured set of genes, cluster 3, shows no GO enrichment for either curve, suggesting it might not be biologically meaningful. Of greatest interest is cluster 4, which is ultimately restricted to D curve and enriched for synaptic organization, suggesting that these neurons are more mature. This discovery fits with the biological process of neural migration which naturally proliferates the deep layers earlier than the upper layers.

### CSN Analysis of Brain Cells from ASD Subjects

To demonstrate how CSN is used to contrast network structure in two populations of cells, we analyze the ASD Brain dataset (16). These data feature single-nuclei RNA-seq (snRNA-seq) data from an ASD study of cortical nuclei assessing 13 cell types and illustrate our results using excitatory neurons layers: L2/3, L4, L5/6, and L5/6-CC. For details of the full analysis pipeline and results, see *SI Appendix*, section 5, Figs. S14–S16, and Tables S5–S7. To illustrate, the results are computed for ASD risk gene, 104 marker genes, and 77 highly expressed genes across cell types (housekeeping genes). We obtain the ASD gene list by intersecting the 992 measured SFARI genes (23) with expressed genes of ASD case and control dataset, and 942 genes remain.

After CSN construction, we compare the differences between control and ASD groups by testing for differences in their networks. We use two types of tests: first, an omnibus test for generic differences, and, second, a targeted test, aimed at identifying high-leverage genes that drive the difference. To test for differences in the distribution of the two classes, we can use a nonparametric distance-based test statistic (DISTp) taken from Matteson and James (26), coupled with a permutation test. This test is highly sensitive to differences in network structure, but a rejection merely indicates that the networks are significantly different. To gain further insights into the network differences, we can utilize the sparse leading-eigenvalue-driven (sLED) test (27). The sLED is designed to detect signal attributable to a modest number of genes in the high-dimensional setting encountered for studies of transcription. To emphasize the contrast with differentially expressed genes, we call these DN genes. The sLED takes as input a method for constructing gene–gene relationship matrices from individual cells, such as the average CSN network or Pearson’s correlation matrix. (Further details can be found in *SI Appendix*, section 6.)

**Fig. 4.**
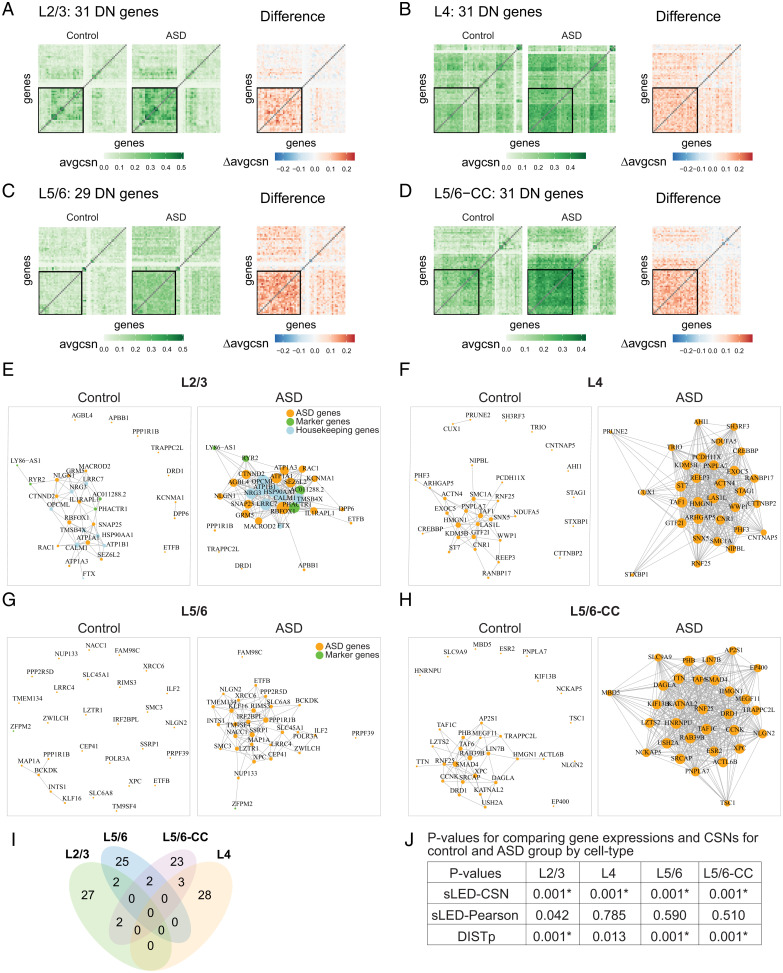
CSN analysis results for ASD Brain dataset. (*A–D*) Heatmaps of average CSNs and the average difference (ASD minus control). Heatmaps display sLED-CSN selected DN genes for each cell type, contrasted with an additional 30 randomly selected genes from 1,123 genes, including 942 ASD genes, 104 marker genes, and 77 housekeeping genes. The black squares delineate the DN genes for each cell type: (*A*) L2/3, (*B*) L4, (*C*) L5/6, and (*D*) L5/6-CC. (*E–H*) Gene networks for DN genes in the excitatory neuron layers. The networks are generated from averaged CSN of control and ASD groups. (*E*) L2/3, (*F*) L4, (*G*) L5/6, and (*H*) L5/6-CC. (*I* and *J*) Contrasting differentially expressed and DN genes for ASD vs. controls. (*I*) Venn diagram of all DN genes for neuron layer cell types. (*J*) *P* values for comparing gene expressions and CSNs for control and ASD groups by cell types. Comparing gene expressions and CSNs for control and ASD groups, *P* values are calculated by sLED–CSN, sLED–Pearson, and DISTp for four excitatory neuron layers: L2/3, L4, L5/6, and L5/6-CC. Asterisk (*) indicates significant differences between two groups after adjusting for multiple testing with 13 cell types.

For each cell type that yielded a significant sLED–CSN difference, we identify the high-leverage genes (defined as the top loading genes that explain 90% of the signal by sLED; see Dataset S2). We call these the “DN genes,” to indicate that they strongly differ between case and control in their coexpression and network structure, but not necessarily in expression. Nearly all the DN genes are ASD genes (*SI Appendix*, Table S8). This suggests that ASD risk gene networks are disrupted in case versus control brains, while marker genes and housekeeping genes are not disrupted. The DN genes identified by sLED–CSN show striking differences in their averaged CSNs (Fig. 4 *A–D*) and also their visualized networks (Fig. 4 *E–H*). Comparing across layers, we find minimal overlap in the DN genes (Fig. 4*I*), suggesting that the network differences are layer specific. The sLED–Pearson test detects no significant differences between control and ASD groups for the neuron layers, in contrast to the sLED–CSN test (Fig. 4*J*).

Restricting to ASD genes, the sLED–Pearson test detects no significant differences between control and ASD groups for any cell type after adjusting for multiple testing, while the sLED–CSN and DISTp tests detect differences in 10 and 6 of the 13 cell types tested, respectively (*SI Appendix*, Table S9 and Figs. S17 and S18). Removal of the DN genes reduces the number of significant differences cell types detected via sLED from 10 to 2, suggesting that they explain much of the difference between the groups (*SI Appendix*, Table S10). For the 10 significant cell types detected by sLED–CSN, we investigate the overlap between the differential expressed (DE) SFARI genes provided in Velmeshev et al. (16) and the sLED–CSN DN genes. There is, surprisingly, no overlap (*SI Appendix*, Table S11), suggesting that most DN genes do not show practically significant differences in expression level between control and ASD groups (Dataset S2); similar conclusions can also be drawn by directly inspecting the marginal expression levels (*SI Appendix*, Fig. S19). Finally, we contrasted layer-specific DN genes identified from among all ASD genes with the remaining ASD risk gene set and found GO enrichment for several intriguing categories, including nervous system development, protein localization, cellular potassium ion homeostasis, and chromosome organization (*SI Appendix*, Fig. S20). Overall, the DN genes offer critical insights about coexpression patterns and how they differ between ASD and control brains.

To further illustrate the applicability of CSN to analyze coexpression patterns, we investigated developing liver cells (*SI Appendix*, section 7, Fig. S21, and Tables S13–S15).

## Discussion

Single-cell gene coexpression networks can yield critical insights into biological processes, but they are challenging to estimate, due to cellular heterogeneity and sparsity of transcript counts. Our proposed solution, locCSN, estimates coexpression networks at the level of the individual cell by sharing strength at the level of the gene pair.

While each CSN is estimated with considerable noise, averaging CSNs over homogeneous cells provides stable estimates of network structure, and this provides insights into how these networks vary by cell type and over developmental epochs. Due to the nonparametric approach, averaged CSN networks provide better estimates of gene block structures than traditional measures.

Subtle differences in network structure can be used to classify cells into subtypes and by cell state. While each CSN is estimated with considerable noise, averaging CSNs over homogeneous cells provides stable estimates of network structure, and this provides insights into how these networks vary by cell type, by cell state, and over developmental epochs. Understanding cell-type-specific gene networks can contribute substantially to our understanding of how biological processes are impacted by disease and disorders (15, 27–29). Just as we can test for differential gene expression, we can test for differential coexpression and aim to detect genes that drive the differences in coexpression. Small changes in gene expression can lead to substantial changes in network structure, ultimately, with large biological effect. To capture this feature, Dai et al. (13) defined “dark” genes as those genes with differences in the degree of connectivity. They are dark because we fail to detect them with transitional differential expression and yet can detect them with CSN analysis. In the same vein, but using a more general definition, we defined DN genes as genes that leverage the differences in network structure. We found that most genes that leverage significant differences of connection between ASD and control groups are missed when simply comparing their expression levels. In a similar setting, using the scHOT algorithm, Ghazanfar et al. (6) identified DN genes in developing mouse liver cells that were not DE genes. Both analyses suggest that traditional analyses of gene expression miss critical signals about gene expression differences across developmental epochs and between phenotypes. CSN offers a powerful framework to discover DN genes.

CRISPR-Cas technologies (30, 31) provide researchers with tools to introduce and assess the effects of many genetic perturbations. For example, Jin et al. (32) used the Perturb-Seq method (31) to introduce dozens of ASD risk genes to developing mice brains and then assessed the impact on the single-cell transcriptome. Because CSN estimates the network of each cell, it is a natural tool for analysis of such perturbation experiments, which target individual cells with distinct perturbations. CSN analysis provides a useful tool for determining how such perturbations impact the network structure of a cell.

Constructing gene networks using the CSN approach is computationally intensive because we compute a test statistics for each pair of genes and each cell; however, a number of approximations can be readily applied to enhance the speed so that CSN can be applied to very large datasets. We found that CSN performed better when applied to metacells, which reduces the number of cells by at least an order of magnitude. It is often natural to reduce the genes under investigation by CSN to a meaningful subset, such as genes previously implicated in genetic risk, genes mapped to critical pathways, or highly variable genes. Restricting the investigation to a subset of genes greatly reduces the computational complexity of CSN analysis, but, more importantly, it can reveal more scientifically interpretable results. By focusing on hundreds of documented ASD risk genes, we were able to identify intriguing network structure in developmental trajectories and changes in network structure between ASD and control subjects. In the literature, several papers have implicated particular cell types, especially neurons and subtypes of neurons, in ASD risk (15, 16, 22, 33–35), but no consensus has been reached. Here we found that gene network structure differed subtly between the developmental trajectories of fetal brain cells for upper-layer and deep-layer excitatory neurons. Specifically, while both trajectories revealed clustering of genes involved in gene expression regulation, only the deep-layer trajectory showed clustering of synaptic genes. Identifying differences in gene networks, both over developmental epochs and between phenotypes, can shed light on the genetic etiology of human phenotypes. For more discussion, see *SI Appendix*, section 10.

## Materials and Methods

### locCSN Method

To compute $z_{xy}^{\left(j\right)}$, the estimated gene–gene relationship of cell *j* for gene pair (*x*, *y*), we first identify the neighborhood and then construct a test statistic. Let $B_{x}^{\left(j\right)}$ and $B_{y}^{\left(j\right)}$ denote one-dimensional bins of cells for genes *x* and *y* centered at the expression levels for cell *j*, with widths *w_x_* and *w_y_*,

$$\begin{array}{l} B_{x}^{\left(j\right)}=\{i:|X_{ix}-X_{jx}|\leq w_{x}\}\;\text{and}\\ B_{y}^{\left(j\right)}=\{i:|X_{iy}-X_{jy}|\leq w_{y}\}, \end{array}$$

with cell counts $n_{x}^{\left(j\right)}=|B_{x}^{\left(j\right)}|$ and $n_{y}^{\left(j\right)}=|B_{y}^{\left(j\right)}|$. Let $B_{xy}^{\left(j\right)}=B_{x}^{\left(j\right)}\cap B_{y}^{\left(j\right)}$ denote the joint window centered at cell *j* with counts $n_{xy}^{\left(j\right)}=|B_{xy}^{\left(j\right)}|$.

The $z_{xy}^{\left(j\right)}$ is represented by a normalized test statistic, $z_{xy}^{\left(j\right)}=\rho_{xy}^{\left(j\right)}/\sigma_{xy}^{\left(j\right)}$, where $\rho_{xy}^{\left(j\right)}$ is a local test statistic for independence of genes *x* and *y*, comparing the joint distribution with the product of the marginals,

$$\rho_{xy}^{\left(j\right)}=\frac{n_{xy}^{\left(j\right)}}{N}-\frac{n_{x}^{\left(j\right)}n_{y}^{\left(j\right)}}{N^{2}},$$

and the normalizing factor $\sigma_{xy}^{\left(j\right)}$ is the asymptotic SD of $\rho_{xy}^{\left(j\right)}$ under the null hypothesis that genes *x* and *y* are independent,

$$\sigma_{xy}^{\left(j\right)}{}^{2}=\frac{n_{x}^{\left(j\right)}n_{y}^{\left(j\right)}(N-n_{x}^{\left(j\right)})(N-n_{y}^{\left(j\right)})}{N^{4}(N-1)},$$

as shown (along with asymptotic normality of $z_{xy}^{\left(j\right)}$ under the null) in Dai et al. (13). We remark that, as a preprocessing step, we fix $z_{xy}^{\left(j\right)}=0$ if the expression of either gene is zero for that cell.

The choice of the window sizes *w_x_* and *w_y_* plays an important role in the performance of the algorithm. Whereas oCSN by Dai et al. (13) uses window sizes equal to a fixed quantile range, we instead choose the window size based on a local SD, which we compute iteratively. Bins $B_{x}^{\left(j\right)}(0)$ and $B_{y}^{\left(j\right)}(0)$ are initialized to include a quantile range [as in Dai et al. (13)], and then, iteratively, we follow

$$\begin{array}{l} w_{x}^{\left(j\right)}(t)=\text{St}.\text{Dev}\;\{X_{ix}:i\in B_{y}^{\left(j\right)}(t-1)\}\\ w_{y}^{\left(j\right)}(t)=\;\text{St}.\text{Dev}\;\{X_{iy}:i\in B_{x}^{\left(j\right)}(t-1)\}\\ B_{x}^{\left(j\right)}(t)=\{i:|X_{ix}-X_{jx}|\leq w_{x}^{\left(j\right)}(t)\}\\ B_{y}^{\left(j\right)}(t)=\{i:|X_{iy}-X_{jy}|\leq w_{y}^{\left(j\right)}(t)\} \end{array}$$

for t=1,… until convergence is achieved (*SI Appendix*, Fig.S1*B*). In practice, if the iterations do not converge, $B_{x}^{\left(j\right)}(1)$ and $B_{y}^{\left(j\right)}(1)$ are used as window sizes. A summary of notation that appears above can be found in *SI Appendix*, Table S12.

### Metacell: Reduce Sparsity of Expressions

For scRNA-seq and snRNA-seq datasets, the expression can be very sparse. In this setting, a direct application of the CSN algorithm fails to discover network structure. It can be advantageous to cluster the data before performing downstream analysis (36); hence, we apply the Metacell algorithm (20) before constructing CSNs. Metacell partitions cells into metacells, defined as disjoint clusters of homogeneous profiles. After applying Metacell to prelabeled cells, we further divide metacells with multiple cell types or subtypes into pure-cell-type metacells. Expression of a metacell is defined as the mean of the cells in the cluster, which alleviates the problem of having zero expression for many genes per cell. The metacells are then treated as cells for the purpose of constructing metacell-specific networks. In this paper, for convenience, CSN refers to either a cell-specific network or metacell-specific network.

### Trajectory-Based Community Detection

The PisCES algorithm (11) is designed to identify cluster structure that varies smoothly over adjacent developmental periods. Following CSN construction for a smooth trajectory, where the cells have been binned by similar pseudotimes, we apply the PisCES algorithm using the average CSN of each bin as input. The time-varying community structure can then be visualized using Sankey plots.

### Runtime of locCSN

The CSN approach can be computationally intensive because we compute a test statistics for each pair of genes and each cell; however, a number of speed ups are possible. By replacing cells with metacells (20), we can reduce the computational complexity substantially. We also provide an approximate CSN calculation by partitioning the outcome space for each pair of genes into a grid. Cells that fall into the same grid yield the same test statistic. With these approximations, CSN can be readily applied to larger datasets with good accuracy (*SI Appendix*, section 8 and Fig. S22). Computational time can also be reduced by limiting the number of genes. While a large number of genes are expressed overall, a relatively modest number of genes are expressed in each cell type, and it is natural to restrict analysis to this subset. Another option is to limit investigation to genes of particular biological interest, such as those implicated in disease or involved in biological processes of interest. Alternatively, to construct a network for a large number of genes (∼1,000), the CSN computation can be applied to each gene pair in parallel.

If only the average CSN is required, cells may be sampled (stratifying within cell type) to reduce computation. To accommodate massive datasets, a possible direction for future work might be to consider fast or vectorizable implementations. This may require precomputation of required quantities; for example, computing the window counts $n_{xy}^{\left(j\right)}$ for all cells *j* might be vectorizable by first constructing a two-dimensional cumulative distribution function of cell counts for a given gene pair.

The examples of runtime against input matrix size are shown in *SI Appendix*, Table S16. Runtimes are measured under Python 3.7.6 [MSC v.1916 32 bit (Intel)]. The runtimes for real data analysis are provided in *SI Appendix*, Table S17.

## Supplementary Material

Supplementary File

Supplementary File

Supplementary File

## Data Availability

All data used in this manuscript are publicly available. The description of dataset for analysis is in *SI Appendix*, section 9. The accession numbers and links of third-party high-throughput sequencing data obtained from the Gene Expression Omnibus and European Bioinformatics Institute databases are listed in *SI Appendix*, Table S18, respectively. All of the code used in these analyses is covered by the MIT License and is available from GitHub: https://github.com/xuranw/locCSN. Previously published data were used for this work (15, 16, 18).

## Change History

January 21, 2022: The byline has been updated.
