# Supplementary material for: Constructing local cell-specific networks from single-cell data
Source: Proc Natl Acad Sci U S A. 2021 Dec 13;118(51):e2113178118. doi: 10.1073/pnas.2113178118 (PMC8713783; doi:10.1073/pnas.2113178118)
Supplement: Supplementary File [file pnas.2113178118.sapp.pdf]

PNAS
www.pnas.org

Supplementary Information for

Constructing Local Cell Specific Networks from Single Cell Data

Xuran Wang, David Choi and Kathryn Roeder

Kathryn Roeder.
E-mail: roeder@andrew.cmu.edu

This PDF file includes:

Supplementary text
Figs. S1 to S22 (not allowed for Brief Reports)
Tables S1 to S18 (not allowed for Brief Reports)
Legends for Dataset S1 to S2
SI References

Other supplementary materials for this manuscript include the following:

Datasets S1 to S2

Supporting Information Text

1. Performance under a correlated bivariate normal

locCSN and oCSN return networks (CSNs) in which each edge is the value of a test statistic that can be used to reject the null of independence between two genes. These networks can be used for two-sample testing, and their average gives the fraction of cells for which the test is rejected, which may be used as an aggregate indicator of nonlinear co-expression.

Fig. S2**a** shows the results of oCSN (1) and locCSN on data simulated from a correlated bivariate normal distribution,

$$\begin{pmatrix} X \\ Y \end{pmatrix} \sim N(\begin{pmatrix} 0 \\ 0 \end{pmatrix}, \begin{pmatrix} 1 & \rho \\ \rho & 1 \end{pmatrix})$$

where $\rho = 0.4$. For this example, the relationship between X and Y is the same for all cells, and thus the ideal test would reject the null of independent X and Y for all cells.

We find that oCSN rejects the independence hypothesis for only 16% percent of the data points, while locCSN shows greater power, rejecting 55%. The lower power of oCSN in this example can be attributed to the choice of a fixed quantile range for the window used to estimate the marginal and joint densities. In particular, in areas of high density, where oCSN's power is lowest, the window becomes extremely small (Fig. S2**b**). As a result, oCSN has good power to detect co-expression only for extreme points, while locCSN has good power over a greater range of expression values. Similar patterns can be found in Fig. S2**c**, which shows that locCSN chooses a constant window size for correlated Gaussian data, and in Fig. S2d, which shows p-values computed by oCSN and locCSN for correlated normal data with ρ ranging between 0.1 and 0.9.

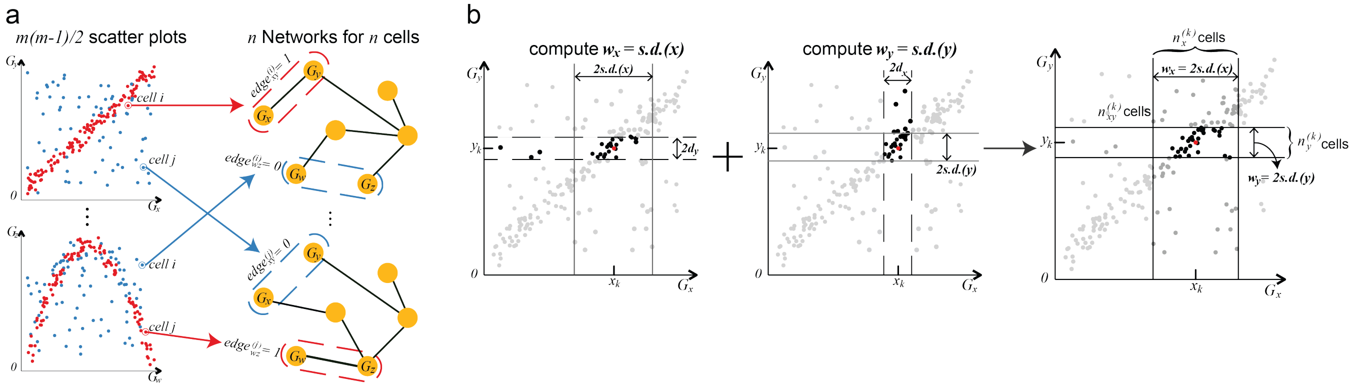

Fig. S1. (**a**) Adapted from Dai et al.(1, Figure 1A). Scatterplots of gene expression levels for gene pairs (x, y) and (w, z), showing regions of high and low density (red and blue) compared to the product of the marginal densities, with corresponding CSN edges or non-edges for these gene pairs highlighted for two cells i and j. The connection between gene pairs are different across cells. (**b**) Standard deviation derived window size for one cell. The first scatter plot shows the quantile derived window for gene y, $w_y = 2d_y$. With this window size, we calculate the standard deviation of gene x expression, using cells within this window size. We take the obtained standard deviation for gene x as the window size for gene x, $w_x = 2s.d.(x)$. Second scatter plot shows the same thing as the first one with swapped x and y. Finally, we use the window size derived from standard deviation for further calculation, that is $w_x = 2s.d.(x)$ and $w_y = 2s.d.(y)$.

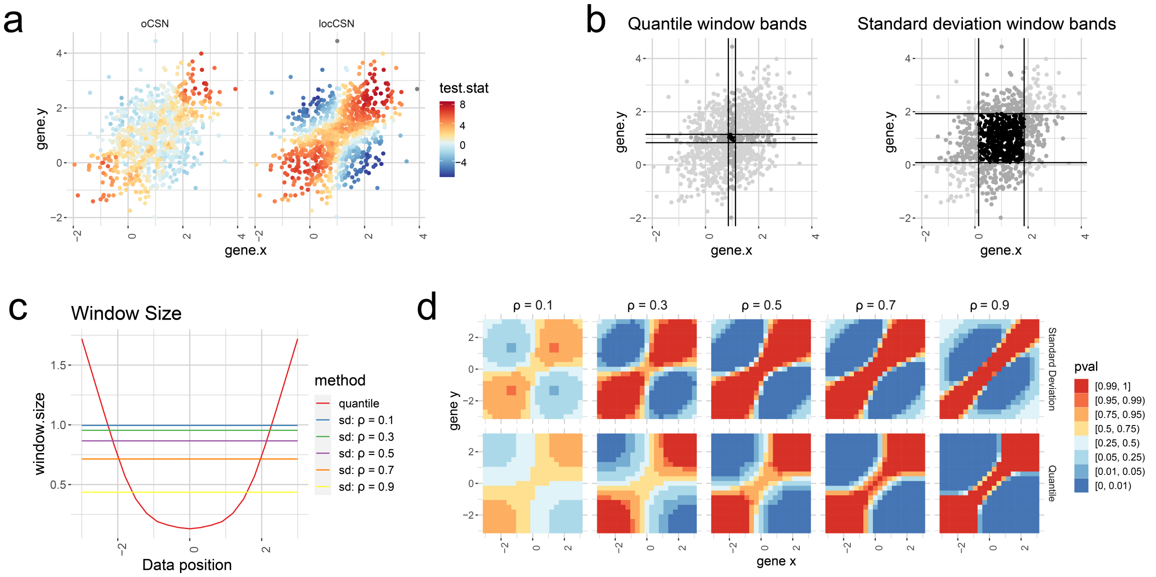

Fig. S2. CSN window size analysis. (**a**) Scatter plot of dataset simulated from normal distribution with $\rho = 0.4$. The left panel is colored by the test statistics calculated from oCSN while the right panel is colored by the test statistics calculated from locCSN. (**b**) Scatter plot of the same dataset simulated from normal distribution. The left panel shows the 10% quantile window bands for a data point in the center. Dark gray shows points selected by gene x band or gene y band. Black shows points selected by both gene x and gene y bands. Light gray indicates points that are not selected by either bands. The right panel shows the scatter plot of the same dataset, window bands are derived from standard deviations. (**c**) Plots of Window sizes derived from quantile and standard deviation for normal distribution with different correlation ρ. The x-axis is the data point position and the y-axis is the window size. Different color shows window sizes derived from quantile or standard deviation under different correlations. (**d**) p-values of test statistic found using quantile or standard devatiation window sizes, under different correlations.

2. Simulations study of CSN parameters

The choice of parameters influence the CSN performance. Using different connection strength and expression levels between genes, we study how different window size and threshold affect CSN performance. We simulated datasets from ESCO (2) with two different settings: 1. True counts without technical noise; 2. Counts with technical noise.

True counts without technical noise. Simulation results are generated from ESCO with a single cell group. There are 200 cells and 100 genes. The code to reproduce this simulation is here: code. The gene-gene correlation matrix exhibits block structure of the form (Fig. S3**a**), or equivalently has off-diagonal entries for the 6 blocks given by the matrix

$$\begin{bmatrix} 0.9 & 0.7 & 0.5 & 0.3 & 0.1 & 0 \\ 0.7 & 0.9 & 0.7 & 0.5 & 0.3 & 0 \\ 0.5 & 0.7 & 0.9 & 0.7 & 0.5 & 0 \\ 0.3 & 0.5 & 0.7 & 0.9 & 0.7 & 0 \\ 0.1 & 0.3 & 0.5 & 0.7 & 0.9 & 0 \\ 0 & 0 & 0 & 0 & 0 & 0 \end{bmatrix},$$

where the first 5 blocks have 15 genes each, and the 6'th block has 25 genes that are independent from all others.

CSN are performed on log-transformed CPM with ESCO simulated read counts data. Fig. S3**c** and **d** shows histograms of the CSN test statistic for gene pairs as a function of their correlation and the choice of window size, which was either a fixed quantile range (as suggested by (1)) of width 5%, 10%, 15%, or 20%; or else initialized to a fixed quantile range and then adapted using locCSN (resulting in "standard deviation window sizes"). We see that the test statistic is shifted for gene pairs that are highly correlated.

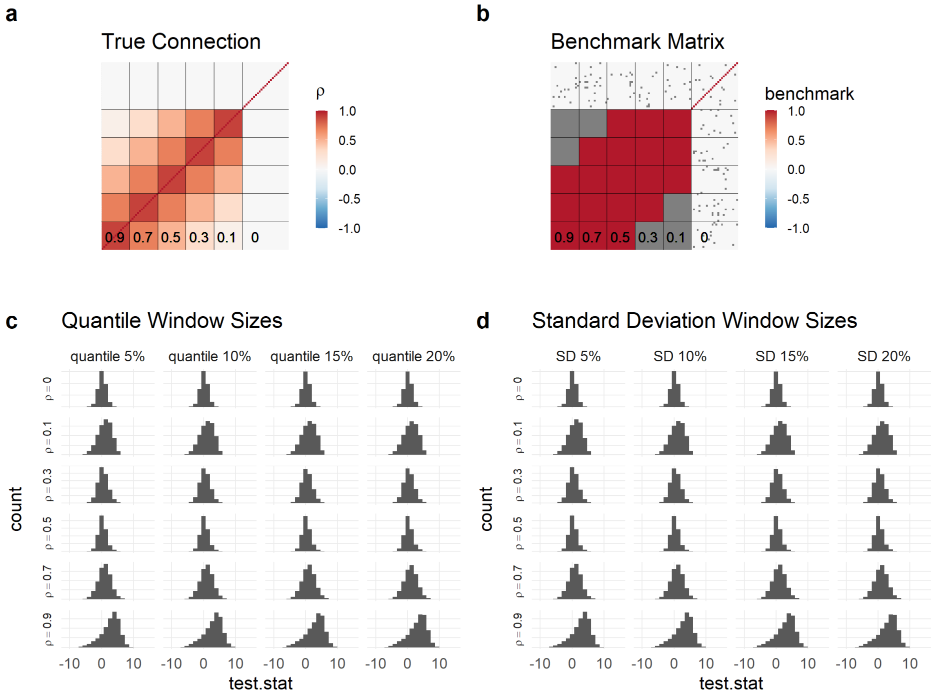

Fig. S3. ESCO simulated single cell expressions without technical noise. (**a**) Heatmap of block structure in gene-gene correlation matrix used for ESCO simulation. (**b**) Gene pairs used for task of classifying pairs with correlation $\rho \geq 0.5$ vs $\rho = 0$. (**c** and **d**) Histogram of CSN test statistics. We exclude genes that are not expressed for all cells. The x-axis shows the CSN test statistics and y -axis is the counts of test statistics. We separate different correlations ρ with rows and different window sizes are shown in columns. (**c**) Histogram for quantile window sizes, 5%, 10%, 15% and 20%. (**d**) Histogram for standard deviation (SD) with starting window size at 5%, 10%, 15% and 20%.

For the same choice of window sizes, Fig. S4**a-c** show ROC and AOC for the task of discriminating gene pairs that are uncorrelated from those whose correlation is ≥ 0.5. 2100 genes pairs were randomly selected for this task, balanced between the two categories. The curves show that for this task, the standard deviation-based window sizes used by locCSN perform better than the fixed quantile range windows proposed by (1). Fig. S4**g** and **h** shows that the average CSN heatmaps using the standard deviation-based window size resemble the original connection matrix (Fig. S3**a**) more strongly that do those using the quantile-based window sizes.

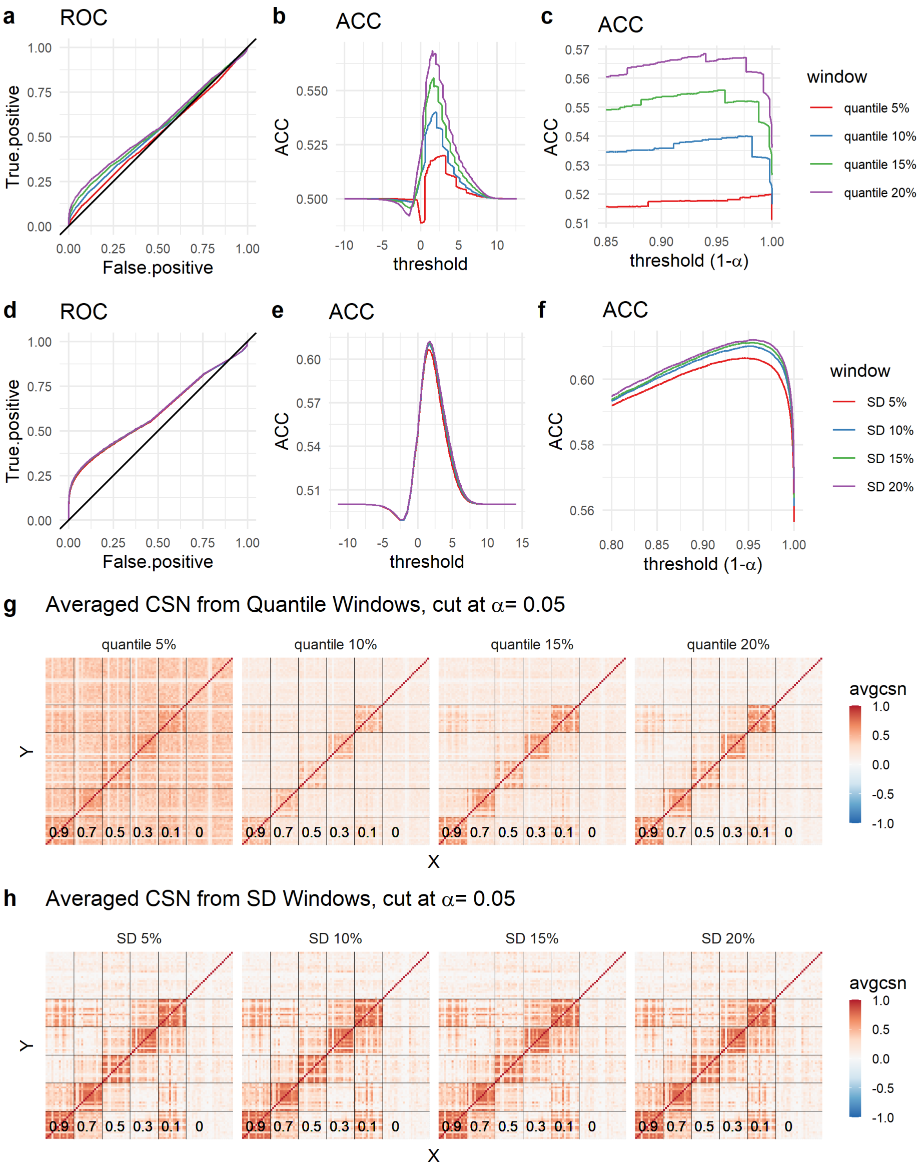

Fig. S4. Evaluation of CSN test statistics from simulated dataset. (**a**-**c**) Evaluations of quantile window sizes. (**a**) ROC curve; (**b**) Accuracy (ACC) curve; (**c**) ACC curve with threshold $Z_{(1-\alpha)}$. The x-axis is $1-\alpha$. (**d**-**f**) Evaluations of quantile window sizes. (**d**) ROC curve; (**e**) ACC curve; (**f**) ACC curve with threshold $Z_{(1-\alpha)}$. The x-axis is $1-\alpha$. (**g**-**h**) Heatmaps of averaged CSN. Each panel shows averaged CSN for a specific window size. The threshold for having edges is $\alpha = 0.05$. (**g**) Heatmaps of averaged CSN for quantile windows; (**h**) Heatmaps of averaged CSN for standard deviation windows.

Counts with technical noise. To better represent single-cell dataset, which are extremely sparse, we use the down-sampling feature in ESCO to produce realistic simulated data. Increasing sparsity weakens the strength of connection in the observed datasets, so we use correlation matrices with stronger values to produce meaningful results. Simulation is again performed by ESCO with 200 cells and 100 genes. We study 2 scenarios:

1. Strong connection: there are 4 blocks of genes, 25 genes each. Within each block, genes are highly correlated with $\rho = 0.95$. Genes from different blocks are independent (Fig. S5**a**);

2. With weaker connection: same as above, but blocks 3 and 4 are not independent, and instead have a weaker correlation $\rho = 0.5$ (Fig. S6**a**).

The parameters for ESCO simulation with down-sampling are set at lib.loc = 7 and alpha_mean = 0.7. lib.loc indicates the overall expression level of the datasets and alpha_mean controls the strength of down-sampling. 40%-50% of the simulated expression are zeros, which approximately corresponds to single-cell RNA-sequencing data with high depth for a particular cell-type. The code to reproduce two simulation scenarios is here: code.

Fig. S5**c**-**e** and S6**c**-**e** show ROC curves and ACC for the task of discriminating gene pairs that are uncorrelated from those with positive correlation. Results show better performance when using locCSN compared to using fixed quantile window sizes.

We also show the heatmap of the average CSN (Fig. S5**f** and S6**f**), thresholded at $\alpha = 0.01$ for strong connection scenario and at $\alpha = 0.05$ for the weaker connection scenario.

The two scenarios both support that standard deviation windows work better than quantile windows in terms of false discovery and accuracy. The simulation also suggests that the choice of threshold also depends on how true connections are defined. If we only consider strong connections (correlation > 0.9) as connected, we can use larger threshold. On the other hand, if we want to include medium strength connections (correlation ≥ 0.5) as connected, we can use the smaller threshold.

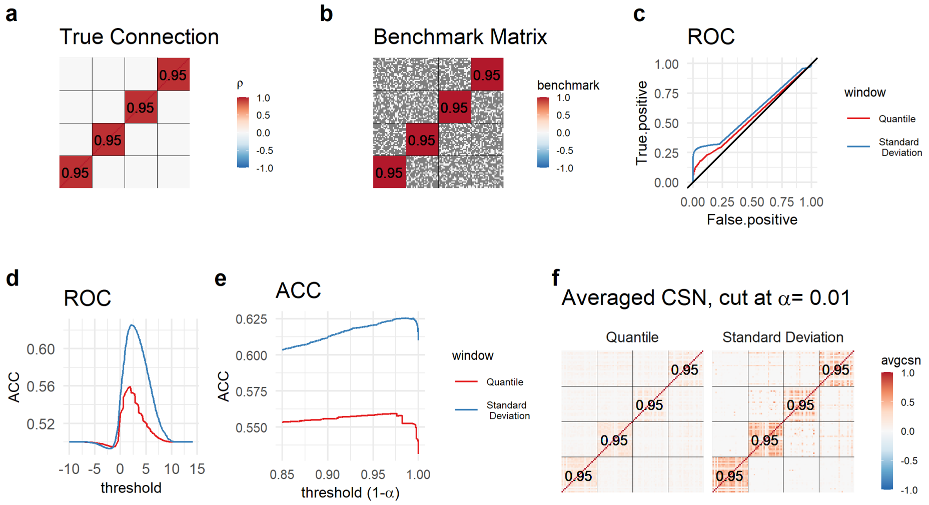

Fig. S5. Evaluation of ESCO simulated dataset with strong connections and technical noise. (**a**) Heatmap of true correlation matrix; (**b**) Gene pairs used for classification task; (**c**-**e**) Evaluation curves for quantile and standard deviation window sizes. (**c**) ROC curve; (**d**) ACC curve; (**e**) ACC curve with threshold $Z_{(1-\alpha)}$. The x-axis is $1-\alpha$. (**f**) Heatmaps of averaged CSN with threshold at $\alpha = 0.01$. Two panels indicate quantile window and standard deviation window.

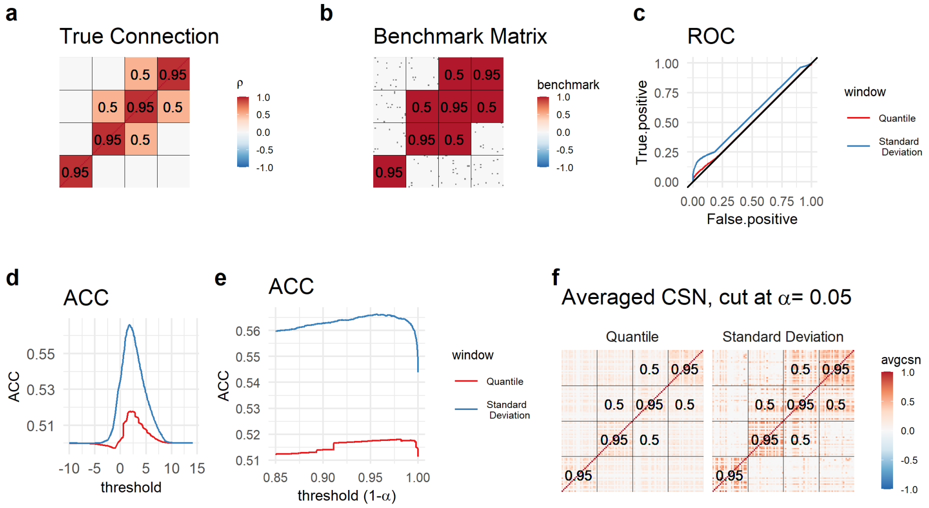

Fig. S6. Evaluation of ESCO simulated dataset with weaker connections and technical noise. (**a**) Heatmap of true correlation matrix; (**b**) Gene pairs used for classification task; (**c**-**e**) Evaluation curves for quantile and standard deviation window sizes. (**c**) ROC curve; (**d**) ACC curve; (**e**) ACC curve with threshold $Z_{(1-\alpha)}$. The x-axis is $1-\alpha$. (**f**) Heatmaps of averaged CSN with threshold at $\alpha = 0.05$. Two panels indicate quantile window and standard deviation window.

Comparison between Pearson's correlation and CSNs. Using the same simulation with technical noise as above, we compare the empirical Pearson's correlation matrix and the averaged CSNs to the true block-structured correlation matrix. CSNs are calculated using standard deviation window size and averaged CSNs are thresholded at $\alpha = 0.01$ and $\alpha = 0.05$. Fig. S7 shows the heatmaps of true and estimated matrices for two scenarios. In both scenarios, averaged CSN identifies the block structure of the true correlation matrix (A), as does the empirical Pearson's correlation matrix.

Surprisingly, in this example the averaged CSN not only identifies the simulated block structure, as would be expected of a useful measure of co-expression, but also estimates the true correlation more accurately than the empirical Pearson's correlation in L_1 norm, as can be seen in Table S1. While the average CSN is not designed to be an estimator of Pearson's

correlation, apparently in this particular simulation the robustness of the averaged CSN to technical noise outweighs its bias in estimating this quantity.

To give an intuitive example where such a phenomenon might possibly occur, consider a simple model where X and Y are random variables with identical marginal distributions (after centering and rescaling), where $Y = X$ with probability p and is generated independently of X otherwise. In this case, it can be seen that the Pearson correlation of X and Y is equal to p, the fraction of data points for which X and Y are not independent – i.e., the fraction that should reject a local independence test analogous to locCSN or oCSN. In such a case, it might be possible that estimating the fraction p of non-independent points could be more accurate than generically estimating the Pearson correlation, particularly if the data has high noise.

Table S1. Distances between true connection matrix and estimated matrices, measured by L_1 norm.

	Strong Connection	With Weaker Connection
Average CSN ($\alpha = 0.05$)	30.73	46.73
Average CSN ($\alpha = 0.01$)	27.67	46.95
Pearson's Correlation	37.66	50.42

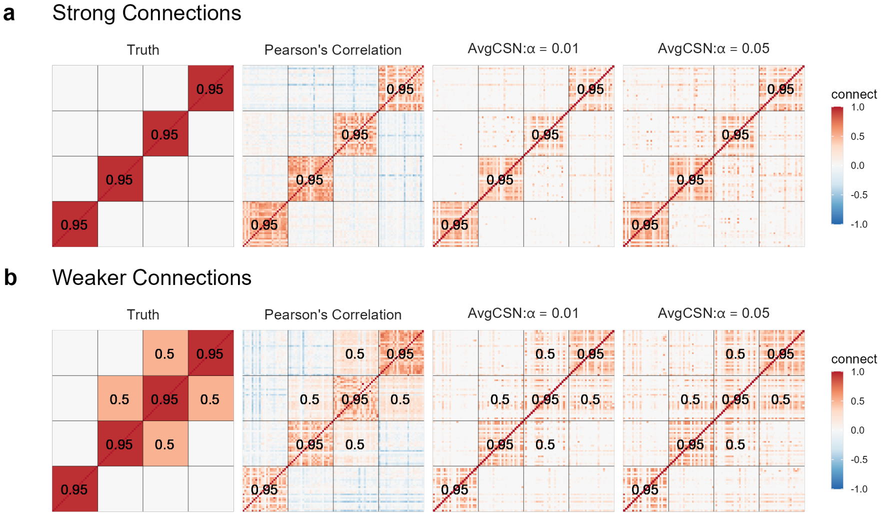

Fig. S7. True correlations and estimates using either empirical correlation or average CSN. The first panel shows true correlations and the following 3 panels shows the estimates. The second panel shows empirical Pearson's correlation. The next two panels are the averaged CSN with threshold $\alpha = 0.01$ and $\alpha = 0.05$. (**a**) Strong connections; (**b**) Weaker connections.

Compare CSN with BigSCale correlation. Next we simulate data from ESCO, with 10000 genes and 2500 cells. We focus on 125 housekeeping genes that are not correlated with each other. After down-sampling the read counts to weaken the signal, we compare BigSCale (3) and locCSN using metacells. With no correlation between genes, we should not detect connections between genes. From 2500 cells, we constructed 158 metacells and on average, there are 15 cells per metacell. The heatmaps of Pearson's correlation of down-sampled and true read counts are shown in the first panel of Fig. S8**f**. The BigSCale correlation shows false positives between genes when there are no connection between genes. By contrast, average CSN with metacells shows no connection between genes.

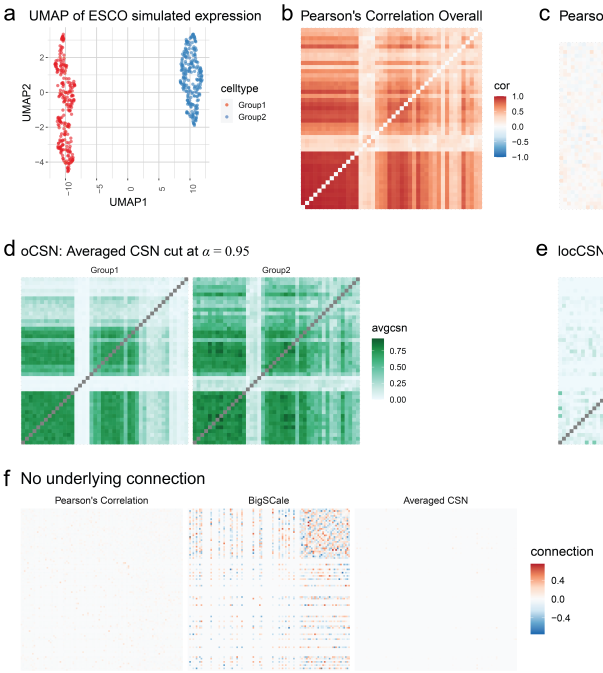

Fig. S8. Network estimation for two simulated cell-types. (**a**) UMAP from ESCO simulated gene expression. (**b**) Heatmap of Pearson's correlations of genes, calculated ignoring cell-types. (**c**) Heatmaps of Pearson's correlations of genes, calculated independently for each cell-type. (d-e) Heatmaps of averaged CSN within cell-type, thresholded by $\alpha = 0.95$ quantile of standard normal distribution. (**d**) oCSN calculated ignoring cell-types. (**e**) locCSN calculated independently for each cell-type. (**f**) For a dataset simulated using ESCO with no correlation between any genes, heatmaps of Pearson's correlation, estimated BigSCale network and averaged locCSN.

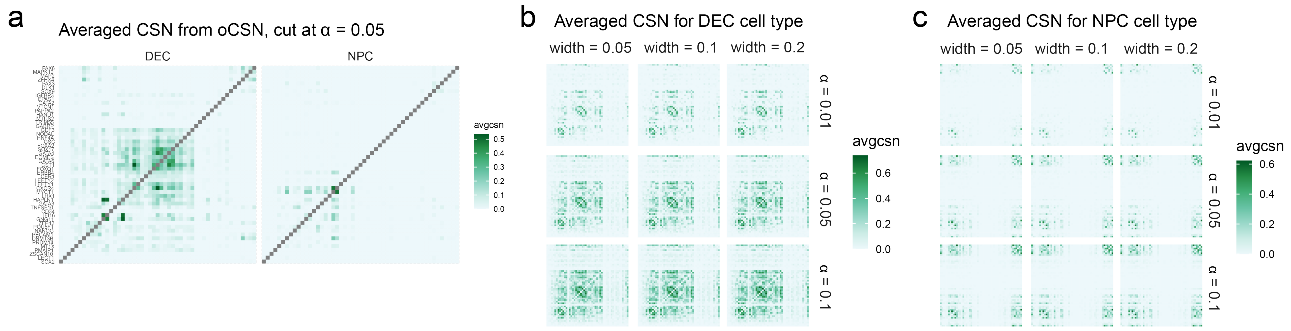

Fig. S9. Heatmap of averaged CSN calculated from Chu et al. dataset. (**a**) Heatmap of averaged CSN calculated from oCSN for DEC and NPC cell-type. The cut-off is $\alpha = 0.05$. (**b**-**c**) Heatmap of averaged CSN calculated from locCSN for DEC and NPC cell-type with different parameters. The column of panels shows different window sizes $= 0.05, 0.1$ and 0.2 while the row of panels shows different cut-off $\alpha = 0.01, 0.05$ and 0.1. (**b**) DEC cell-type. (**c**) NPC cell-type.

3. CSN trajectory analysis of Developing Cortex Atlas dataset: data processing

For the human brain cortex atlas data, we focus on 10 cell types from 4 samples for analysis, specifically the 6 neuron cell types: ExDp1, ExDp2, ExM, ExM-U, ExN and IP, plus 4 radial glia and progenitors (P) cell-types: vRG, oRG, PgS and PgG2M. Metacells are constructed sample by sample and within a subtype. Based on Fig. 3, ExDp1 and ExDp2 cell-types are combined as ExDp and the 4 radial glia and progenitors cell-types as P for further analysis (ExDp are subsequently partitioned at a later stage of analysis). Table S2 shows the number of cells and metacells for 7 major cell-types: P, IP, ExN, ExM, ExM-U and ExDp.

Prior to CSN construction along the curve, we generate metacell bins based on pseudotime of the curve within each cell-type. Each bin contains around 800 metacells, which are relatively homogeneous; however, for each cell-type, some metacells are deemed outliers based on their pseudotime scores. We retain metacells whose pseudotime are within 2 standard deviations of the mean within the bin. These are the cells that will be utilized for CSN construction. The number of bins used for each cell-type, and number of metacells (before and after outlier screening) are listed in Table S3. Since there are only 58 metacells for ExM-U in the leftmost curve, we ignore this cell-type for the D-Curve analysis. This cell-type properly belongs in the U-curve analysis. Table S3 shows the overlapping of metacells for the two curves. P metacells are shared across both curves. IP and ExN metacells are largely shared between the two curves. The split in trajectories occurs during development of the ExM cell-type, which show considerably less overlap of metacells between the two curves.

Table S2. Number of cells and metacells in cell-types.

Cell-type	P	IP	ExN	ExM	ExM-U	ExDp
Number of cells	4204	2150	9995	9822	1756	2205
Number of metacells	720	574	2759	2415	424	271

Table S3. Number of metacells in two curves for 6 cell-types. Before and after removal of pseudotime outliers. The overlap between two curves

Curves	D-Curve						U-Curve				
Cell type	P	IP	ExN	ExM	ExM-U	ExDp	P	IP	ExN	ExM	ExM-U
Number of metacells	720	574	2451	1604	58	265	720	569	1913	1189	335
Remaining metacells	720	559	2373	1488	0	262	720	531	1804	1097	324
Overlapped metacells	720	521	1779	422	0	0	720	521	1779	422	0
Number of bins	1	1	3	2	0	1	1	1	3	2	1

		D-Curve				
		cluster 1	cluster 2	cluster 3	cluster 4	Total
U-Curve	cluster 1	52^*	0	5	0	70
	cluster 2	2	73^*	9	30	160
	cluster 3	0	9	86^*	13	127
	Total	59	103	111	47	

Table S4. Number of genes in gene communities of D-curves and U-curve. The rows indicate 3 gene clusters from U-curve and the columns show 4 gene clusters from D-curve. The "total" row and column show the total number of genes in each gene cluster. The intersect between U-curve gene clusters and D-curve gene clusters are shown in each row and clusters. The sum of number of overlapped genes does not sum to the total number of genes because some genes are not clustered in both trajectories. * indicates two clusters in D-Curve and U-Curve have a strong overlap with each other.

Figure S10(a)(b) shows average CSN networks as heatmaps for bins ExN_pt2 and ExN_pt3 in D-curve and U-curve, along with flows showing the movement of genes between the communities as taken from the Sankey plots in Fig. 3. Community 1 can be seen to have greater density of connections; this is also shown in Figure S10(c)(d), which shows the density of connections for all 8 bins comprising the trajectory, also reveals differences in connection density for communities 2, 3, and 4, which may not be visible in the heatmaps shown in S10(a)(b).

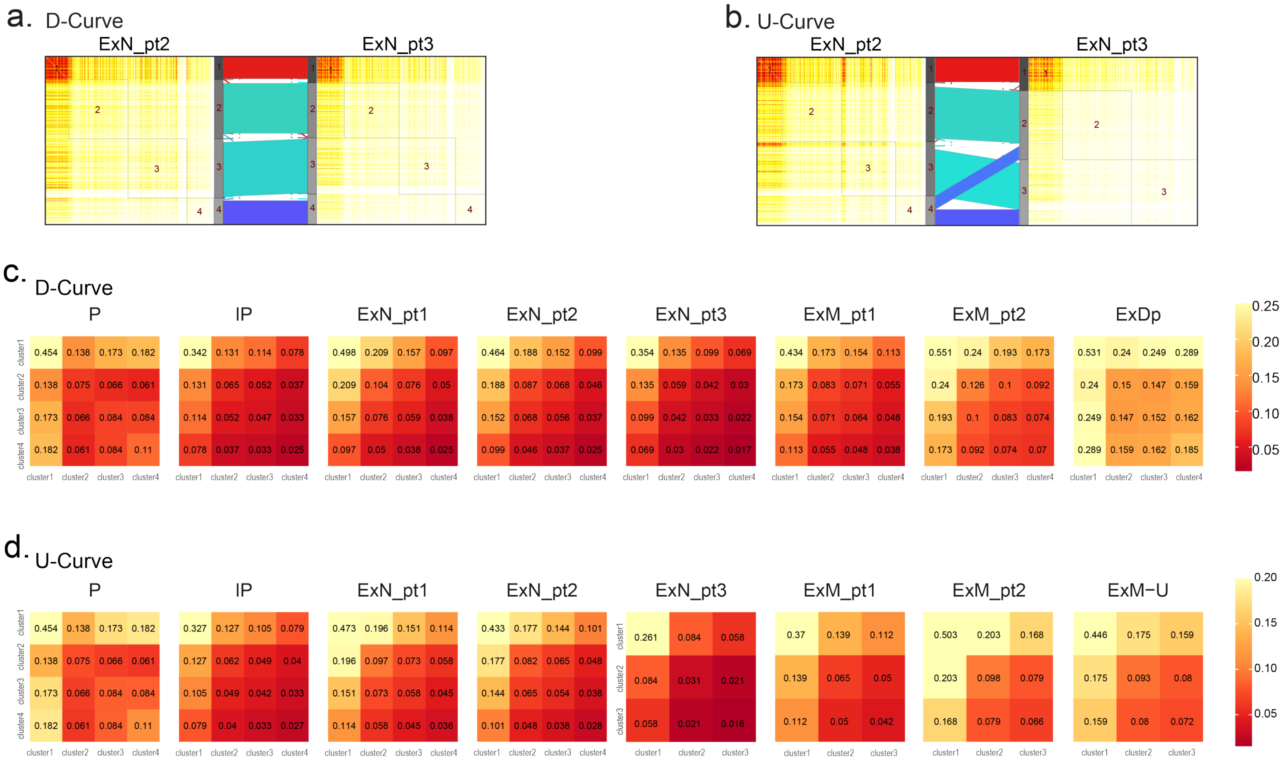

Fig. S10. (**a** and **b**) Sankey plots of gene flows from ExN_pt2 to ExN_pt3, with heatmaps showing average CSNs for both stages (**a**) D-Curve. (**b**) U-Curve. (**c** and **d**) Heatmaps showing average connections within and between each cluster throughout entire developmental trajectory. (**c**) D-curve. (**d**) U-curve. Color scales were chosen to show contrast between non-dense clusters 2, 3, and 4.

Figure S11 shows boxplots of averaged metacell gene expression. Clusters 2, 3, and 4, which were separated into clusters based on their differing levels of co-expression as shown in Fig. S10, have lower levels of expression compared to cluster 1.

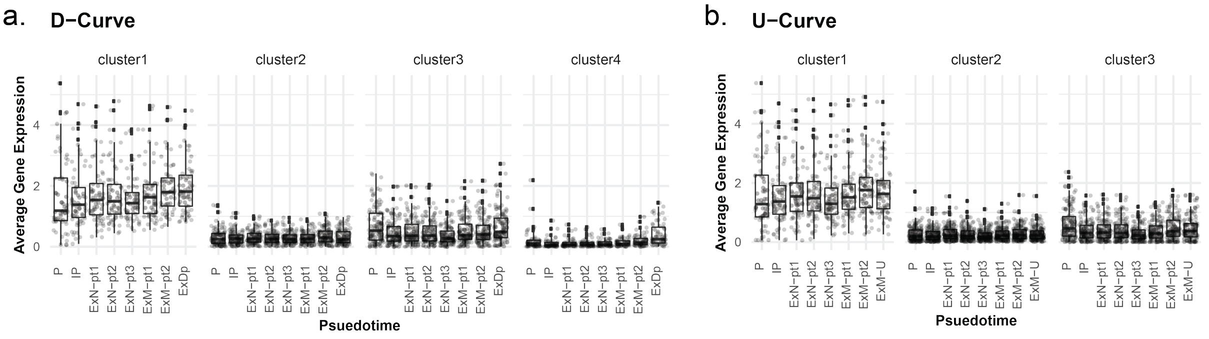

Fig. S11. Boxplots of averaged metacell gene expression for 8 pseudotime bins in the final 4 and 3 clusters for the D-curve (**a**) and U-curves (**b**), respectively. The x-axis shows pseudotime bins and y-axis shows the averaged expression.

Fig. S12 shows that the WGCNA algorithm fails to detect gene modules in either the ExDp or ExM-U cell-types, when Pearson's correlation matrices for gene expression is used as input to WGCNA. There are 441 genes and 262 metacells for ExDp and 440 genes for 324 metacells for ExM-U. This result contrasts with the module structure discovered using CSN data as input to PisCES (Fig. S10).

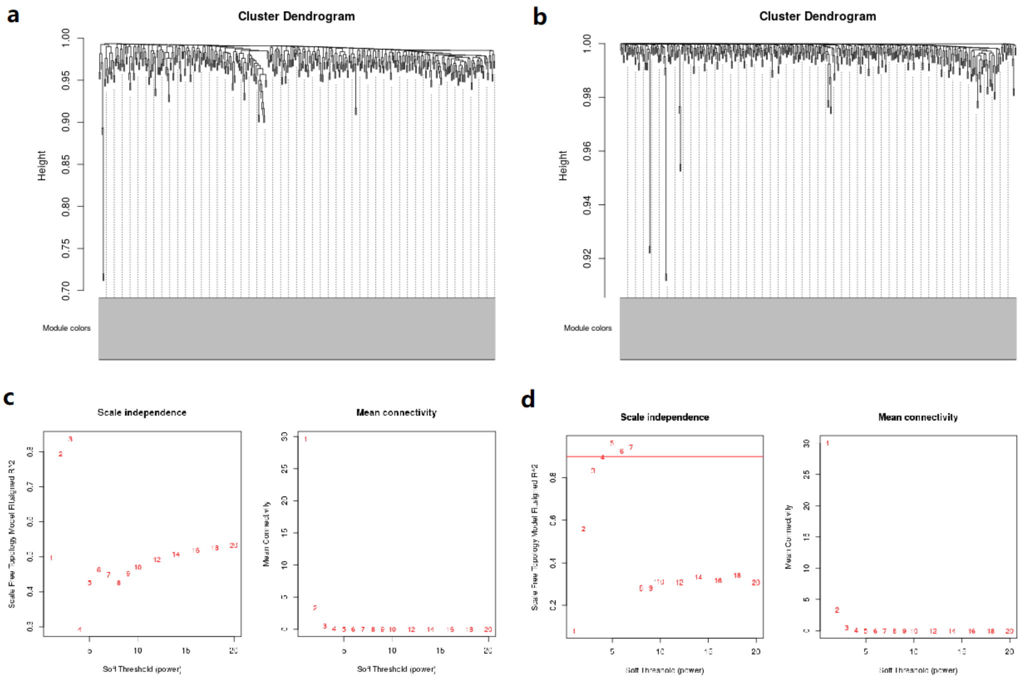

Fig. S12. Gene modules generated from Pearson's correlations using WGCNA. (**a**) ExDp from D-curve; (**b**) ExM-U from U-curve. (**c**) power selection plots for ExDp from D-curve: power = 2. (**d**) Power selection plots for ExM-U from U-curve: power = 4.

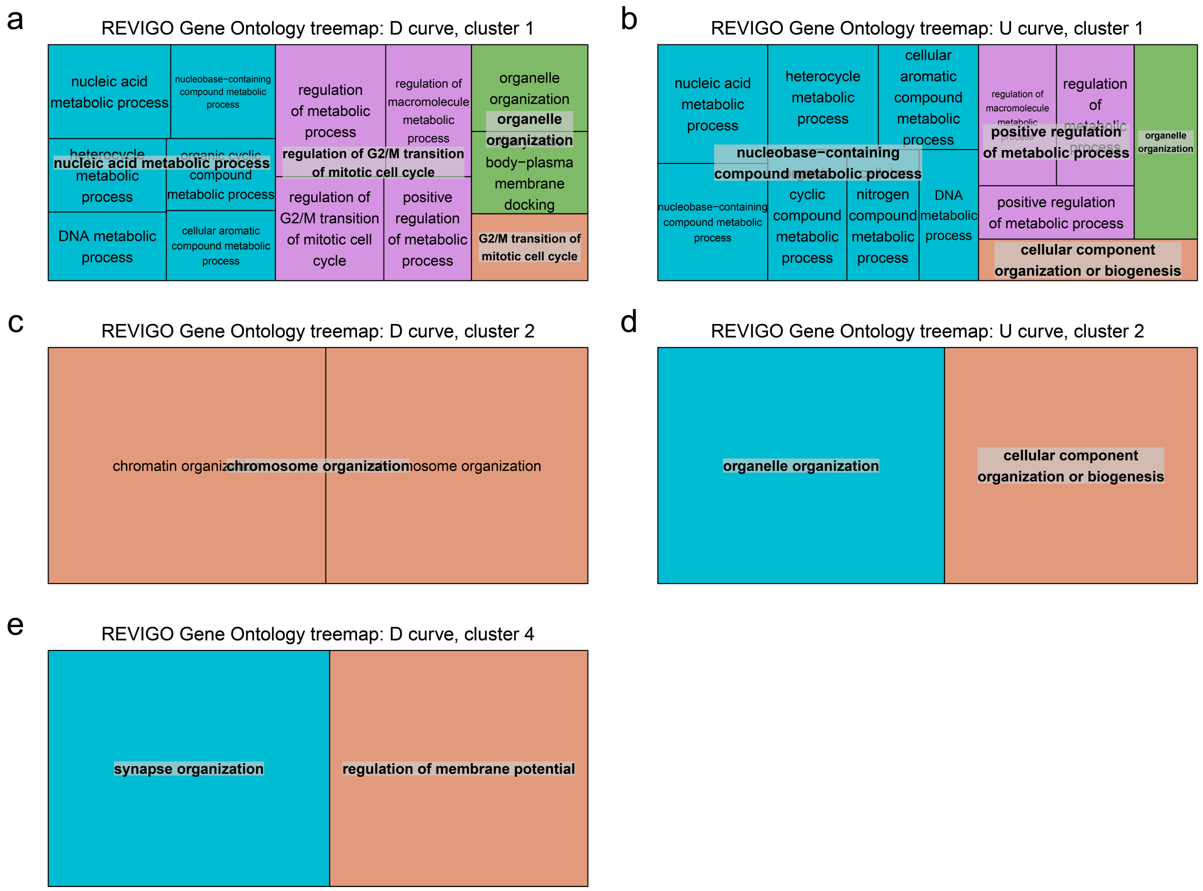

Fig. S13. Revigo treemap GO terms for gene clusters in D-curve and U-curve. (**a**) D-curve cluster1 (dense cluster); (**b**) U-curve cluster1 (dense cluster); (**c**) D-curve cluster2; (**d**) U-curve cluster2; (**e**) D-curve cluster4.

4. Gene Ontology (GO) term treemap

The Gene Ontology (GO)(4) describes our knowledge of the biological domain with respect to three aspects: Molecular function. Cellular component and Biological process. In this paper, we focus on biological process. The p-values for GO terms indicate enrichment of the selected gene list in a GO category. Using all ASD genes as the gene universe, an FDR adjusted $p < 0.01$ was considered to be statistically significant. GO treemaps are created by REVIGO with default setting (5) and the areas in GO treemaps indicate the absolute log10 p-value of GO terms.

5. CSN analysis of ASD Brain dataset: data processing

The ASD brain dataset (6) consists of single-nuclei RNA-seq measured from 41 samples (22 ASD and 19 controls) from human brains. The authors classify the ASD brain dataset to 17 cell-types: fibrous astrocytes (AST-FB), protoplasmic astrocytes (AST-PP), endothelial (End), parvalbumin interneurons (IN-PV), somatostatin interneurons (IN-SST), SV2C expressing interneurons (IN-SV2C), VIP expressing interneurons (IN-VIP), upper-layer excitatory neurons (L2/3), layer 4 excitatory neurons (L4), deep layer cortico-subcortical excitatory projection neurons (L5/6), Deep-layer cortico-cortical excitatory projection neurons (L5/6-CC), microglia (Mic), immature neurons (Neu-mat), neurogranin expressing neurons I (Neu-NRGN-I), neurogranin expressing neurons II (Neu-NRGN-II), oligodendrocytes (Oligo) and oligodendrocyte precursor cells (OPC) (6). For our analysis, we merge some cell subtypes together depending on whether the cell-types are distinct in the tSNE plot (Fig. S14**a** and S15). For instance, the AST* cell-types are merged into one cell-type, while the L* cell clusters are distinct and analyzed individually (Fig. S14**b**).

To circumvent challenges due to sparse counts, which are especially prevalent in single-nuclei RNA-seq data, we cluster similar cells and form metacells (7). To avoid batch effects, metacells are created within a sample and cell-type (Table S7). We merged AST-*, IN-*, L* and Neu* together as broad cell-type for the summary of the number of metacells (Table S6). The number of metacells in each cell-type are shown in Table S7. Within a cell-type, some metacells exhibited heterogeneity that was poorly delineated into clusters. For each metacell within a cell-type, we constructed CSNs using the nearby 100 metacells from UMAP plot of the combined ASD and control cells (Fig. S16).

For broad cell-type AST, In, L and Neu, more than one original cell-type is included within the broad cell-types. We then determine, based on heterogeneity of cells, whether to analyze the cells within a broad cell-type or within a more refined cell-type. Numbers of metacells in each original cell-type are presented in Table S7. From the UMAP and tSNE plot, we decide to analyze AST as a broad cell-type without division. We divide IN broad cell-type into 2 major cell types (IN-SV2C + IN-VIP) and (IN-SST+IN-PV), the L broad cell type is divided into 4 cell-types: L2/3, L4, L5/6, and L5/6-CC and Neu into 2 cell-types Neu-mat and (Neu-NRGN-I + Neu-NRGN-II) (Fig. S15). Cell-types can be analyzed at different levels depending on heterogeneity of the cells and available sample sizes. The original data were partitioned into 17 original cell-types, which spanned 8 broad cell-types. Based on separation of clusters, we performed our analysis on a compromise partition resulting in 13 cell groups, which we refer to as cell-types hereafter. The relationship between the various partitions of cell clusters is shown in Table S5. Metacells for cell-types are shown in Table S7.

Table S5. Relation between 17 original cell-types, 8 broad cell-types and the 13 cell-types we feature in our analysis.

Broad cell-type	AST		End	IN				Oligo	Mic
Cell-type	AST		End	IN-PV,SST		IN-VIP,SV2C		Oligo	Mic
Original cell-type	AST-FB	AST-PP	End	IN-PV	IN-SST	IN-SV2C	IN-VIP	Oligo	Mic
Broad cell-type	Neu			L				OPC	
Cell-type	Neu-mat	Neu-NRGN		L2/3	L4	L5/6	L5/6-CC	OPC	
Original cell-type	Neu-mat	Neu-NRGN-I	Neu-NRGN-II	L2/3	L4	L5/6	L5/6-CC	OPC	

Table S6. The number of metacells for samples and broad cell-types. * indicates the ASD sample while others are the control samples.

**sampleID**	**1**	**2**	**3**	**4***	**5***	**6***	**7**	**8**	**9***	**10***	**11***	**12***	**13**	**14**	**15***	**16**	**17***	**18***	**19***	**20**	**21**	**22***
**AST**	4	18	13	17	18	24	5	13	12	31	8	15	21	10	21	15	11	17	33	29	16	23
**End**	1	10	9	3	5	4	4	4	4	3	2	7	23	8	1	2	1	5	5	1	11	5
**IN**	13	36	30	33	26	23	29	11	15	27	14	22	60	19	7	19	17	16	34	27	27	38
**L**	9	58	69	49	50	22	15	22	30	55	25	39	148	61	14	36	40	15	45	44	48	65
**Mic**	2	2	6	2	1	11	2	5	2	9	9	8	9	0	2	10	3	4	7	5	9	2
**Neu**	13	43	34	69	65	8	26	50	25	14	29	33	100	24	5	29	15	13	28	19	133	33
**Oligo**	11	28	54	40	7	24	1	66	19	5	28	22	21	18	5	16	27	3	7	51	17	8
**Opc**	7	18	20	15	17	11	9	9	6	22	10	10	39	8	13	18	17	8	17	14	11	19
**sampleID**	**23***	**24**	**25***	**26***	**27**	**28**	**29**	**30**	**31***	**32***	**33***	**34**	**35**	**36**	**37***	**38***	**39**	**40***	**41***		**Ctl**	**ASD**
**AST**	18	29	32	17	14	14	14	25	17	28	26	6	6	5	16	5	21	17	19	**AST**	278	425
**End**	3	4	8	1	4	11	3	1	3	4	3	1	2	1	4	2	2	1	1	**End**	102	75
**In**	27	21	40	21	16	31	19	18	18	32	18	26	25	17	33	11	27	6	13	**In**	471	491
**L**	47	58	84	46	30	42	44	22	24	85	49	36	48	19	30	20	33	39	63	**L**	842	936
**Mic**	3	9	15	2	13	8	9	9	0	3	7	3	2	0	2	0	2	1	1	**Mic**	105	94
**Neu**	29	62	75	28	13	40	20	17	21	27	10	28	22	18	19	8	13	16	27	**Neu**	704	597
**Oligo**	7	50	49	5	16	8	72	14	1	8	23	45	13	1	3	7	2	9	3	**Oligo**	504	310
**Opc**	14	12	26	9	10	8	12	14	11	12	13	16	9	8	16	9	19	14	11	**Opc**	261	300

Table S7. Number of metacells for 13 cell-types.

#metacell	AST	End	IN-VIP,SV2C	IN-PV,SST	L2/3	L4	L5/6	L5/6-CC	Mic	Neu-mat	Neu-NRGN	Oligo	OPC
Control	278	102	238	253	414	238	107	177	105	244	353	504	261
ASD	425	75	265	206	358	211	109	164	94	235	469	310	300
Total	703	177	503	459	772	449	216	341	199	479	822	814	561

Table S8. Number of DN genes that are ASD genes, markers genes and housekeeping genes (HKG).

Leverage genes	L2/3	L4	L5/6	L5/6-CC		DN	L2/3	L4	L5/6	L5/6-CC
Total	92	94	106	89		Total	31	31	29	31
ASD	60	89	102	87		ASD	19	31	28	31
Marker	10	4	3	2		Marker	4	0	1	0
HKG	22	1	1	0		HKG	8	0	0	0

Table S9. p-values from sLED-CSN, sLED-Pearson and DISTp for all 13 cell-types. $*$ indicates significant difference (p-value < 0.0038) after adjusted for multiple testing. The leverage genes are the non-zero entries of the sparse leading eigenvector. We only provide DN genes for significant cell-types, corresponding to genes that explain 90% of the variability among the leverage genes.

P-values	AST	End	IN-VIP,SV2C	IN-PV,SST	Mic	Oligogo	OPC
sLED-CSN	0.001*	0.474	0.001*	0.001*	0.125	0.001*	0.001*
sLED-Pearson	0.024	0.243	0.376	0.654	0.680	0.315	0.030
DISTp	0.023	0.815	0.002	0.010	0.294	0.051	0.001*
leverage genes	122	103	95	69	83	80	76
DN genes	26	NA	26	26	NA	28	27
P-values	L2/3	L4	L5/6	L5/6-CC	Neu-mat	Neu-NRGN	
sLED-CSN	0.001*	0.001*	0.001*	0.001*	0.022	0.002*	
sLED-Pearson	0.089	0.861	0.564	0.348	0.341	0.174	
DISTp	0.001	0.039	0.001*	0.001*	0.256	0.002*	
leverage genes	79	112	89	87	146	110	
DN genes	27	26	24	27	NA	26	

Table S10. p-values from sLED-CSN after removing DN genes. The removal is for the 10 cell-types with significant signal in the original analysis (Table S9). $*$ indicates significant difference (p-value < 0.005) after adjustment for multiple testing.

Cell-type	AST	IN-PV,SST	IN-VIP,SV2C	Neu-NRGN	Oligo	OPC	L2/3	L4	L5/6	L5/6-CC
DN	26	26	26	26	28	27	27	26	24	27
P-value	0.074	0.023	0.006	0.474	0.109	0.001*	0.001*	0.050	0.012	0.040

Table S11. Numbers of ASD differentially expressed (DE) genes and DN genes

cell-type	AST	IN-VIP,SV2C	IN-PV,SST	Oligogo	OPC
SFARI DE genes	12	18	11	0	1
DN genes	26	26	26	28	27
cell-type	L2/3	L4	L5/6	L5/6-CC	Neu-NRGN
SFARI DE genes	16	17	1	2	5
DN genes	27	26	24	27	26

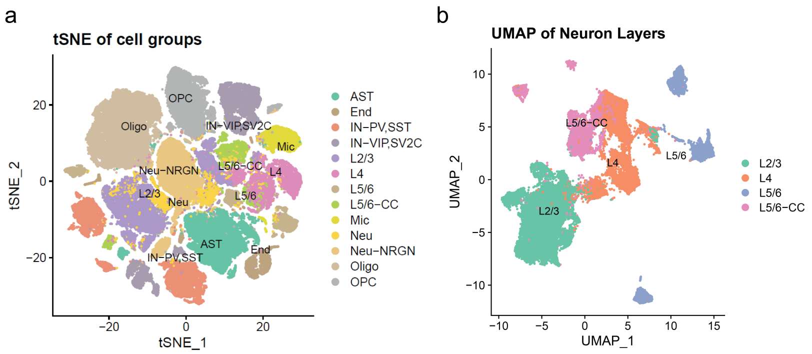

Fig. S14. Dimension reduction of brain cells. (**a**) tSNE plot of all brain cells colored by cell-types. (**b**) UMAP plot of Neuron Layers(L) cells colored by 4 cell-types.

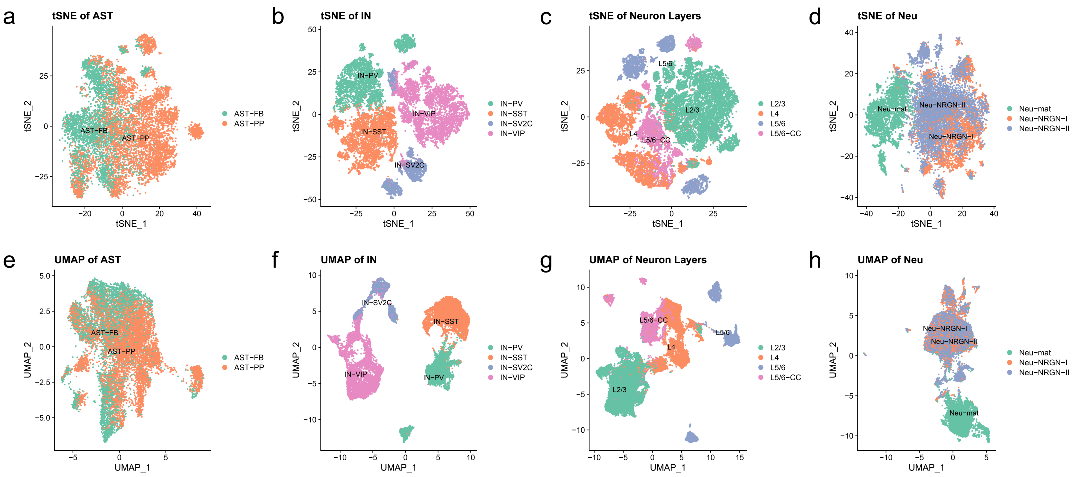

Fig. S15. Dimension reduction of 4 broad cell-types, colored by the author defined cell-type labels. (**a**) astrocytes(AST); (**b**) interneurons(IN); (**c**) neuron layers(L); and (**d**) neurons(Neu);

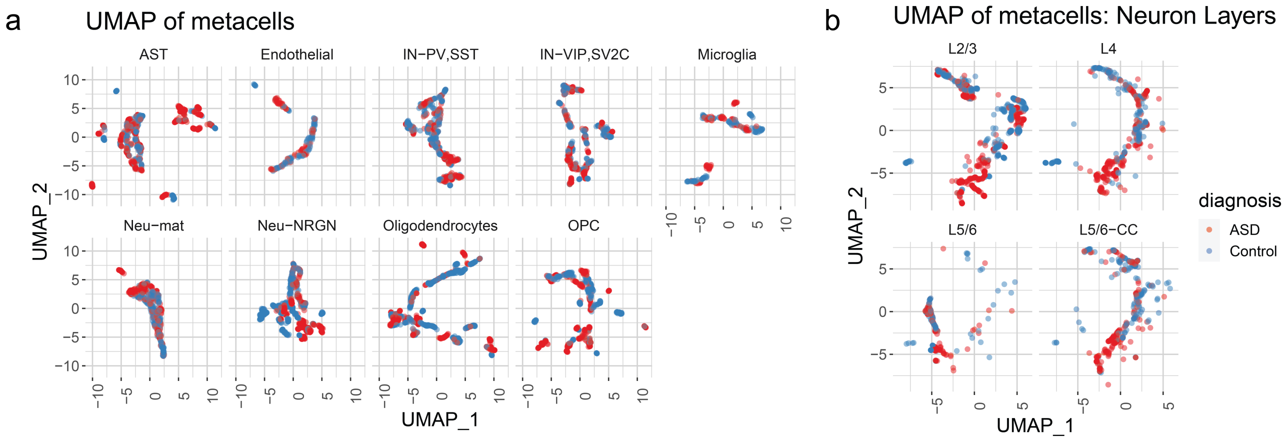

Fig. S16. UMAP of metacell expressions for 13 cell-types.

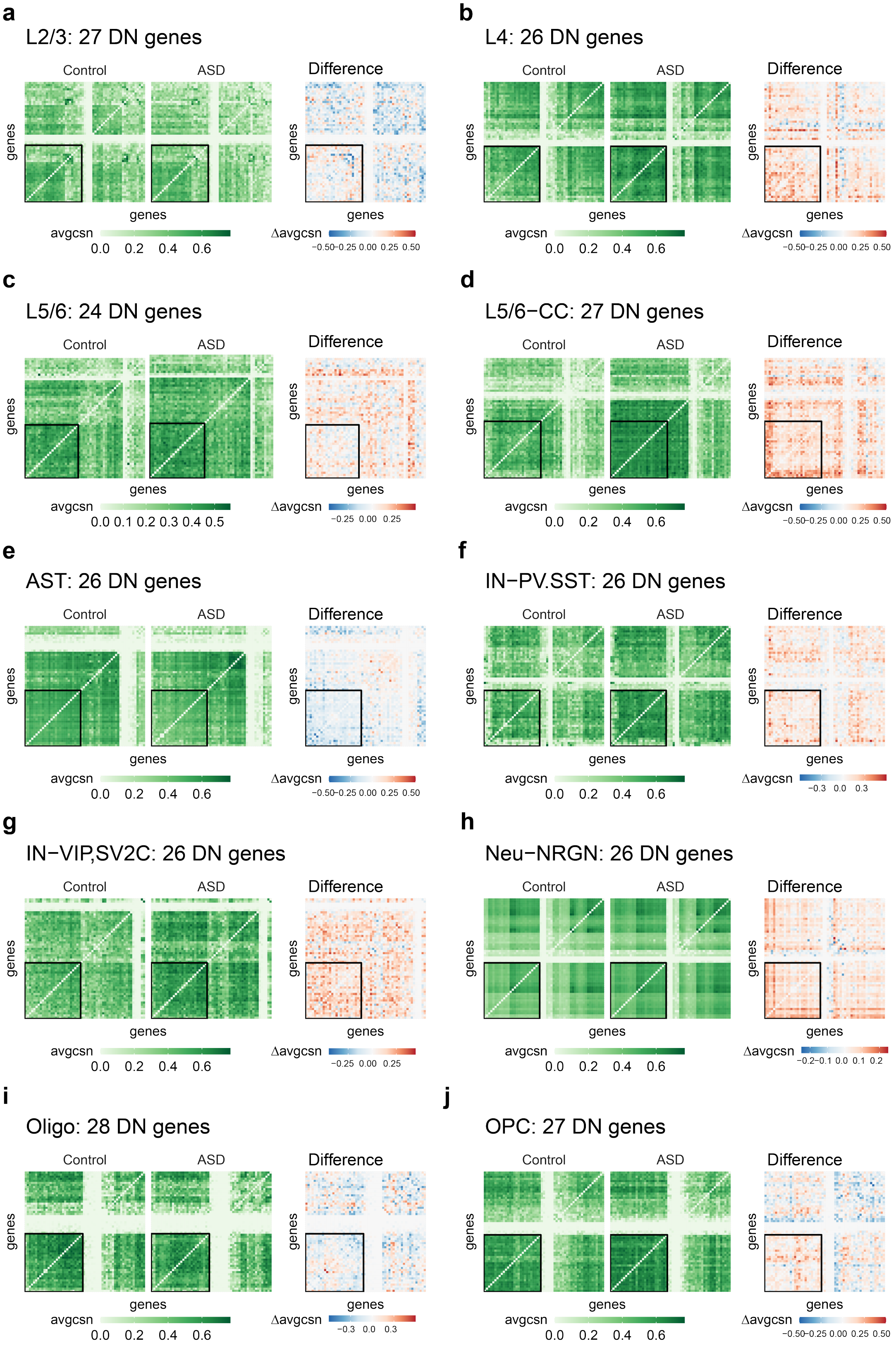

Fig. S17. Heatmaps of average CSNs and difference of average CSN between Control and ASD samples. The heatmaps display sLED-CSN DN genes and an additional 30 randomly selected genes from 942 ASD genes. Genes are ordered for each cell-type for display. The DN genes are outlined in black. The green heatmaps show the averaged CSN for control and ASD groups and the red/blue heatmaps show the difference between averaged CSN between control and ASD groups (ASD group minus Control group). (**a**) L2/3; (**b**) L4; (**c**) L5/6; (**d**) L5/6-CC; (**e**) AST; (**f**) IN-PV,SST; (**g**) IN-VIP,SV2C; (**h**) Neu-NRGN; (**i**) Oligodendrocytes; (**j**) OPC.

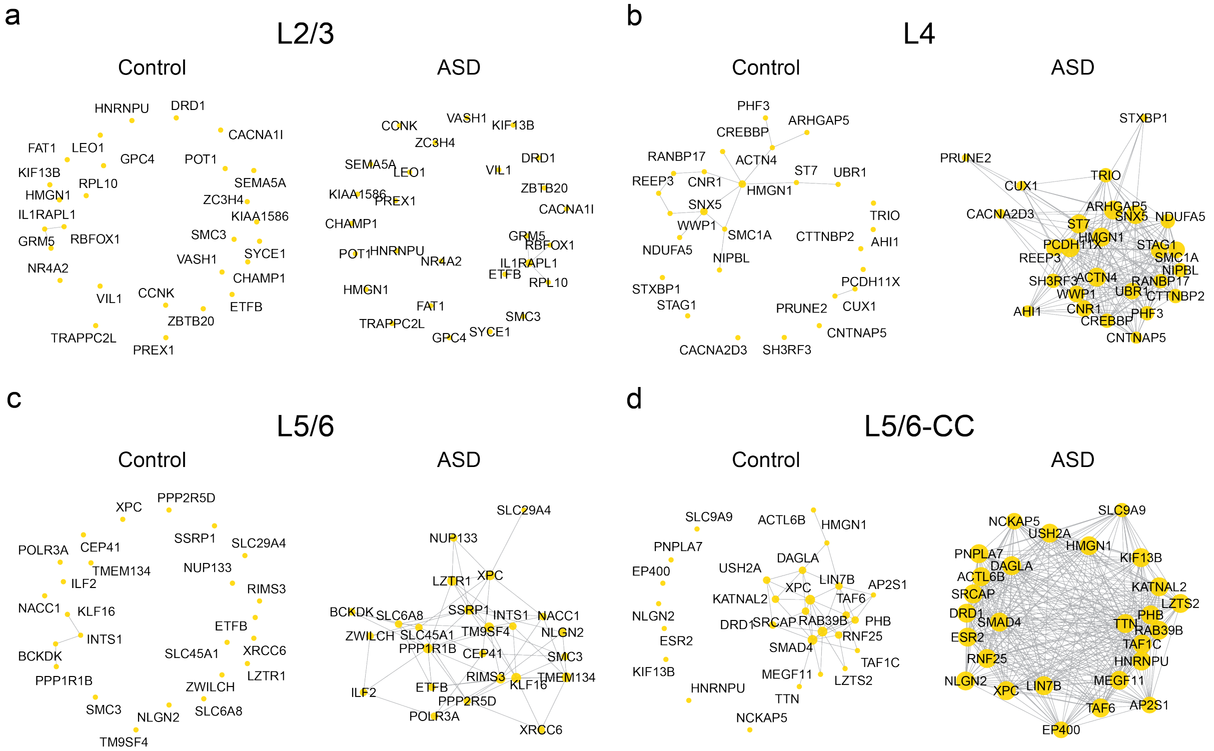

Fig. S18. Gene networks for DN genes in the excitatory neuron layers. The networks are generated from averaged CSN of control and ASD groups: (**a**) L2/3; (**b**) L4; (c) L5/6 ;and (**d**) L5/6-CC.

In general, DN genes tend to have relatively large variance and DE genes have relatively large mean; and for some cell-types ASD samples are significantly more variable than control samples.

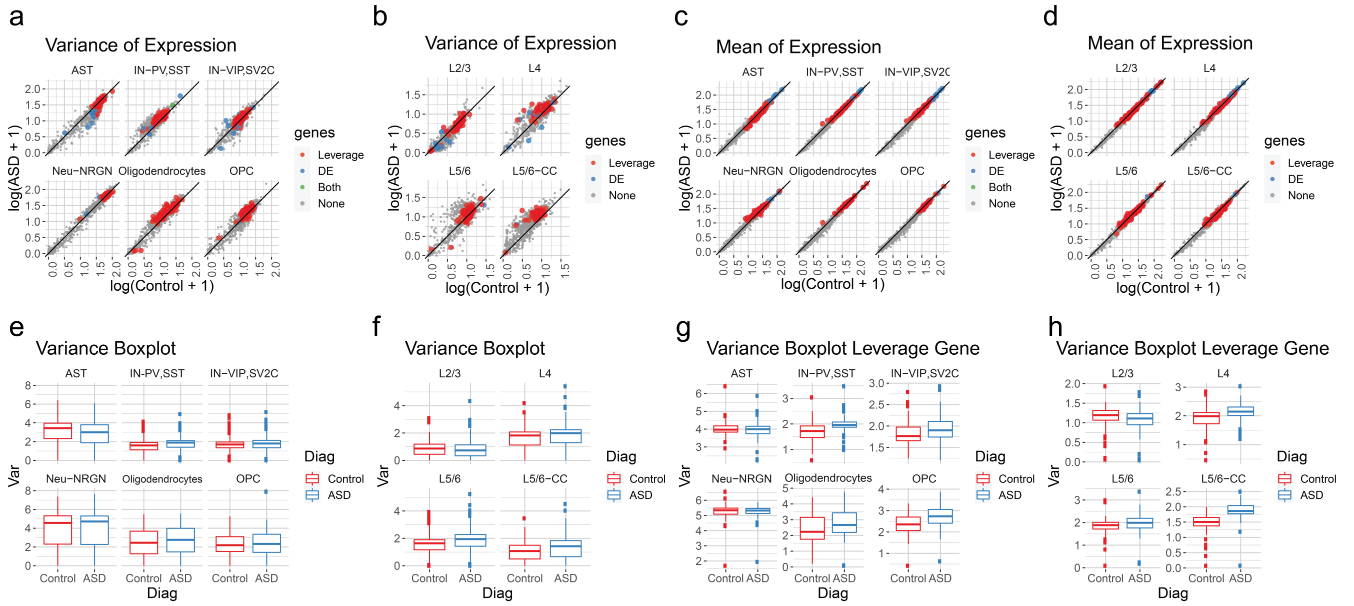

Fig. S19. Variance and mean of gene expression of the 942 SFARI (Simons Foundation Autism Research Initiative) genes in the 10 cell-types with significant sLED-CSN signal. (**a**-**b**) Scatter plots of the variance and mean of gene expression, with x-axis showing the log-transformed values from control group and y-axis from ASD group. The genes are displayed using red and blue to indicate differential network (DN) genes and differential expressed (DE) genes, respectively. (**c**) Boxplots of the variance of gene expression for 942 genes, with red and blue denoting control and ASD, respectively. (**d**) shows the same information for DN genes only.

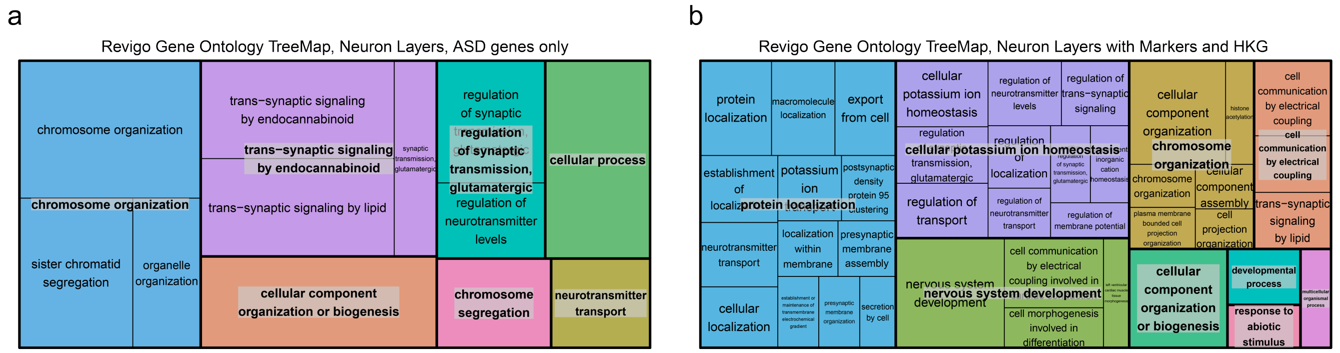

Fig. S20. Revigo treemap GO terms for DN genes from 4 Neuron Layers. (**a**) DN genes selected from ASD genes generated CSNs; (**b**) DN genes selected from ASD and negative control genes (marker genes and housekeeping genes (HKG)).

6. Two sample testing

Given i.i.d. samples of expression levels, the computed CSNs for a set of cells are exchangeable, and hence permutation testing can be used to test for differences in CSN distribution. For this purpose, we suggest two types of tests: first, an omnibus test for generic differences, and second, a targeted test, aimed at identifying high leverage genes that drive the difference.

DISTp: Test CSN differences between groups. Each cell's adjacency matrix can be represented as a vector by converting matrices into vectors, resulting in N_1 sample vectors from class 1 and N_2 sample vectors from class 2. Let $V_1^{(1)}, \ldots, V_{N_1}^{(1)}$ denote the vectorized adjacency matrices from class 1 and $V_1^{(2)}, \ldots, V_{N_2}^{(2)}$ denote the same from class 2. The test statistic Q is a scaled q-norm divergence measurement, with $q \in (0, 2)$ recommended(8), and is given by

$$\begin{aligned} Q(\mathbf{V}^{(1)}, \mathbf{V}^{(2)}; \alpha) =& \frac{2}{N_1 + N_2} \sum_{i=1}^{N_1} \sum_{j=1}^{N_2} |V_i^{(1)} - V_j^{(2)}|^q \\ & - \frac{N_1 N_2}{N_1 + N_2} \binom{N_1}{2}^{-1} \sum_{1 \le i < i' \le N_1} |V_i^{(1)} - V_{i'}^{(1)}|^q \\ & - \frac{N_1 N_2}{N_1 + N_2} \binom{N_2}{2}^{-1} \sum_{1 \le j < j' \le N_2} |V_j^{(2)} - V_{j'}^{(2)}|^q, \end{aligned}$$

with p-value calculated by permutation test.

sLED: Identify differential network genes. The sLED test relies on the same principles as Sparse Principal Component Analysis (SPCA), and was originally proposed for the difference in the Pearson's correlation matrices of the two classes (sLED-Pearson). Here we instead propose using the difference in the average CSN as the test input (sLED-CSN). Given N_1 CSN adjacency matrices from class 1 and N_2 from class 2, denoted by $A_1^{(1)}, \ldots, A_{N_1}^{(1)}$ and $A_1^{(2)}, \ldots, A_{N_2}^{(2)}$, let D denote the difference between the average CSN for each class, so that $D = \bar{A}^{(1)} - \bar{A}^{(2)}$. Then D can be used as the input to sLED, in which case the test statistic is computed from the spectrum of D. Additionally, the test also identifies a small cluster of leverage genes corresponding to the non-zero entries of the sparse leading eigenvector. The differential network genes are the ones that explain 90% of the variability among the leverage genes. These are candidate genes that have altered co-expression structure between the two groups. As with DISTp, the p-value of the test statistic is determined by permuting samples among cell classes.

Table S12. Notations and Definitions

Variable	Definition
N, N_1 and N_2	number of cells (total, class 1 and class 2).
i, j	index of cell: cell i and cell j.
G	number of genes.
x, y	index of gene: gene x and gene y.
w_x, w_y	width of window for gene x and y.
X_{jx}	gene expression of cell j and gene x.
$B_x^{(j)}$, $B_y^{(j)}$, $B_{xy}^{(j)}$	one-dimensional bins for gene x at the expression level for cell j, with window w_x; for gene y at expression level for cell j with window w_y; $B_{xy}^{(j)}$ is the joint window.
$n_x^{(j)}$, $n_y^{(j)}$ and $n_{xy}^{(j)}$	number of cells in bins $B_x^{(j)}$, $B_y^{(j)}$ and $B_{xy}^{(j)}$.
$\rho_{xy}^{(j)}$	local test statistics for independence of genes x and y.
${\sigma_{xy}^{(j)}}^2$	asymptotic standard deviation.
t	iteration in standard deviation window size calculation.
$z_{xy}^{(j)} = \rho_{xy}^{(j)}/\sigma_{xy}^{(j)}$	normalized test statistics.for gene pair (x, y) and cell j.
A_j, $A_j^{(1)}$	Estimated adjacency matrix for cell j and for cell j in class 1.
$a_{xy}^{(j)}$	Entry of A_j, gene pair (x, y) and cell j. $a_{xy}^{(j)} = 0$ or 1.
α	Standard normal tail quantile.
$Z_{(1-\alpha)}$	reverse CDF (cumulative distribution function) of standard normal at $1 - \alpha$.
$\boldsymbol{V}^{(1)}$, $V_i^{(1)}$	vectorized adjacency matrix for class 1 and for cell i in class 1.
q	q-norm for DIST-p distance.
D	the differences between average CSN for each class, $D = \bar{A}^{(1)} - \bar{A}^{(2)}$.

7. CSN analysis of liver dataset

The dataset is from Ghazanfar et al. (9), which contains 447 cells that have been classified based on 3 developmental branches: Cholangiocyte (Cc), Hepatoblast(Hb) and Hepatocyte (Hc). Specifically, as Hb cells mature, the trajectory splits into two mature types: Hc and Cc. For analysis, we selected marker genes using Seurat (140 for Cc, 113 for Hb and 119 for Hc), the highly variable genes(HVG) provided by the scHOT analysis (9), and highly expressed genes (HEG). In addition to those genes, we included genes that are known for molecular regulation of hepatic architecture (10). The number of genes of each category are shown in Table S13.

Table S13. Number of genes for analysis from Liver dataset.

	Marker genes	HVG	HEG	Regulatory genes	Total
# genes	372	67	90	124	620

The expression of marker genes for cells from the 3 branches indicates that Cc has well defined marker genes that are quite different from the other two branches, whereas the markers for Hb and Hc show a smooth evolution in expression across cells, which were ordered by pseudo time (Figure S21). These results suggests that we should construct CSNs for Cc as a set, but for Hb and Hc we should pool the cells together and then compute CSN for each cell within this population of cells. Finally we wish to test if the gene networks for Cc, Hb and Hc differ. We use sLED to perform this test. With sLED-CSN all three pairwise comparisons are highly significant. With sLED-Pearson the comparisons are marginally significant, but the p-values are each smaller and the comparison between Cc and Hb does not survive the multiple testing correlation indicating CSN yields a more powerful test than Pearson's (Table S14). Notably there is no intersect between regulatory genes and sLED-CSN selected leverage genes.

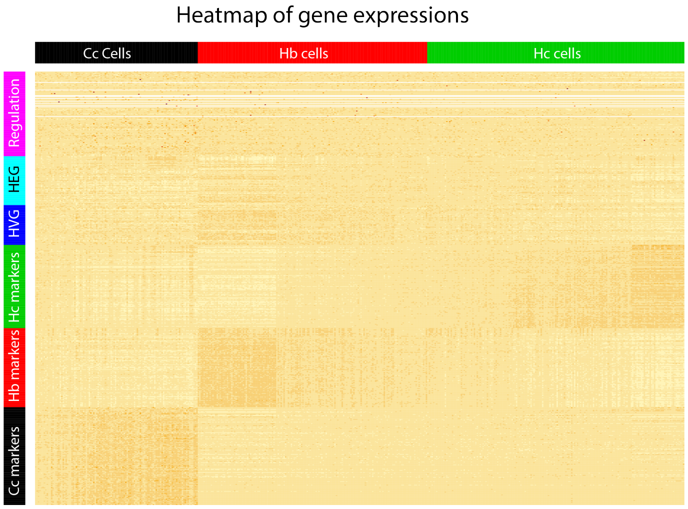

Fig. S21. Heatmap of gene expression of 3 branches from Liver data. Rows correspond to genes: marker genes for Cc (black), Hb (red), Hc (green), HVG (navy), HEG (cyan) and Regulation genes (magenta); and columns for branches, Cs (black), Hb (red) and Hc (green).

Table S14. Table for sLED analysis p-values.

sLED-CSN	Cc	Hb		sLED-Pearson	Cc	Hb
Hb	0.001	.		Hb	0.042	.
Hc	0.001	0.001		Hc	0.004	0.016

Table S15. Table of number of leverage genes and DN genes.

Leverage genes	Cc-Hb	Cc-Hc	Hb-Hc		DN genes	Cc-Hb	Cc-Hc	Hb-Hc
Marker genes	54	51	82		Marker genes	18	17	15
HVG	2	1	0		HVG	0	0	0
HEG	11	11	9		HEG	3	3	0
Regulatory genes	0	0	0		Regulatory genes	0	0	0
Total	59	58	86		Total	18	18	15

8. Runtime of locCSN

The runtime of locCSN are provided below. Three different settings are simulated by ESCO (2) with different sizes of expression matrices (Table S16). For a large set of genes, for example Setting 3 with 1000 genes, parallel computing is recommended to speed up the process of generating CSNs. We also speed up our algorithm by an approximate CSN calculation, which partitions the outcome space for each pair of genes into a grid. Cells that fall into the same grid yield the same test statistic (called fuzzy). With these approximations CSN can be readily applied to large datasets with good accuracy Fig. S22.

Table S16. Runtime of locCSN. Python 3.7.6 [MSC v.1916 32 bit (Intel)]

	Setting 1	Setting 2	Setting 3	Setting 4
Number of genes	100	100	1000	10
Number of cells	200	500	200	10000
Number of expressed entries	13114	31065	39572	42978
Runtime	322.59s	1020.744s	NA	1653.764 s
Speed-up	186.96s (fuzzy)	691.62s (fuzzy)	2260.15s (parallel)	234.230s (fuzzy)

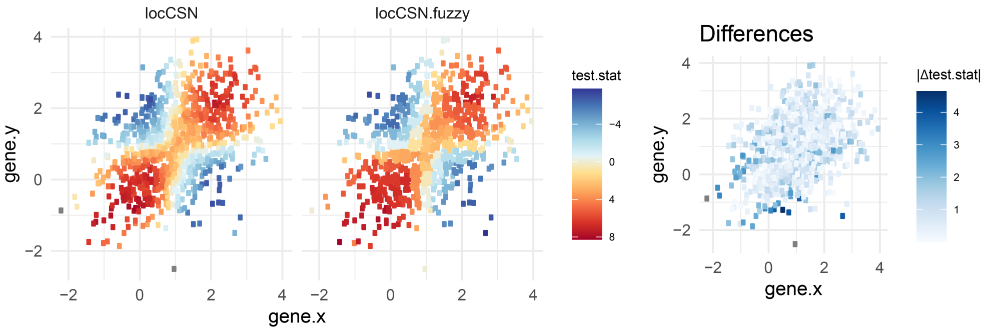

Fig. S22. Comparison of test statistics from locCSN and its grid based (fuzzy) approximation. With the same simulation setting in Fig. S2**a**, we simulate bivariate normal distribution with $\rho = 0.4$. The left panel is colored by test statistics calculated from locCSN while the right panel is colored by test statistics from locCSN fuzzy approximate.

Here we include the runtime for the real analysis. In our package locCSN: https://github.com/xuranw/locCSN, we also include Matlab version and the real data analysis are performed with Matlab R2016a (9.0.0.341360) 64-bit (glnxa64). Except for Chu et al. dataset, other dataset are calculated with parallel computing. On average, the converged pairs of genes will reach convergence after 8.5 iterations (95%:[5, 13] iterations).

Table S17. Runtime of locCSN for real data analysis.

Datasets	Chu et al. dataset	Brain Cortex Atlas	ASD Brain
# genes	51	444	942
# cells or metacells	138 (DEC), 173 (NPC)	Table S3	Table S7
Runtime	27.39s (DEC), 13.08s (NPC)	55136.87s (D-curve), 40387.26 (U-curve)	6950.674s (average)
Speed-up	No	Parallel	Parallel

9. Data summary

Chu et al. dataset. Chu et al.(11) includes 1018 cells and seven cell-types. This dataset contained the cells of human embryonic stem cell-derived lineage-specific progenitors. The cell-types including H1 embryonic stem cells, H9 embryonic stem cells, human foreskin fibroblasts (HFF), neuronal progenitor cells (NPC), definitive endoderm cells (DEC), endothelial cells (EC) and trophoblast-like cells (TB) were identified by fluorescence-activated cell sorting (FACS) with their respective markers. 9600 genes are obtained per cell on average.

Developing Cortex Atlas dataset. Polioudakis et al.(12) includes cells from mid-gestational human cortex (17-18 gestational weeks). These data are derived from 33,986 cells from germinal zones (ventricular zone [VZ], subventricular zone [SVZ]), developing cortex (subplate [SP] and cortical plate [CP]) separated before single cell isolation. Using Drop-seq technology the average reads per cell was 52,000. Expression for 1049 genes was detected per cell.

Autism Spectrum Disorder (ASD) Brain dataset. Velmeshev et al.(6) includes snRNA-seq data from an ASD study, which collected 105 thousand nuclei from cortical samples taken from 22 ASD and 19 control samples from subjects between 4 and 22 years old. Samples were matched for age, sex, RNA integrity number, and postmortem interval.

Table S18. Data summary of single cell data for analysis. * **The code to reproduce this dataset is here: code.**

Datasets	ESCO Synthetic	Chu et al.	Brain Cortex Atlas	ASD Brain
References	Tian et al.(2020) (2)	Chu et al.(2016) (11)	Polioudakis et al.(2019) (12)	Velmeshev et al.(2019) (6)
Tissue	NA	Human Embryonic Stem Cells	Human fetal Brain Cortex	Human brain
# cell	2000	1018	25,013	104,559
# cell-types	2	7	16	18
# genes	100	16,619	35,543	41,202
# genes for analysis	30 markers	51 developmental markers	444 expressed SFARI ASD genes	942 expressed SFARI ASD genes
Data Availability	Github*	GSE75748	Website	PRJNA434002

10. Data Pre-processing Discussion

Log2 transformed CPM datasets are preferred for locCSN analysis and are used for all simulations and real data analysis in this paper. As mentioned in the Discussion section, we found that CSN performed better when applied to metacells, which reduces the number of cells by at least an order of magnitude. It is often natural to reduce the genes under investigation by CSN to a meaningful subset, such as genes previously implicated in genetic risk, genes mapped to critical pathways, or highly variable genes. Restricting the investigation to a subset of genes greatly reduces the computational complexity of CSN analysis, but more importantly, it can reveal more scientifically interpretable results. For example, we focus on 51 developmental genes that are suggested by Chu et al.(11). The choice of cell type is also important for locCSN analysis. For mature cells, it is natural to cluster them by cell types. It is only for developing cells that a trajectory is the better choice. For instance, fetal brain cell types do not plot in distinct clusters in UMAP and they are more naturally ordered by pseudotime. A user would need to make this decision, but it is not a difficult one for a scientist to make in context of their study. To avoid batch effect, we create metacells for each subject, which reduced the effects from first moment shifts of the data. But when comparing cases and controls, we suggest aggregating all metacells/cells to perform locCSN to avoid confounding by phenotype.

SI Dataset S1 (Supplementary_Developing_Cortex_Atlas.xlsx)

Developing Cortex Atlas data results. Tab1: Genes in 4 clusters of D-curves; Tab2: Genes in 3 clusters of U-curves; Tab3: List of expressed ASD genes; Tab4: GO terms for D-curve cluster 1; Tab5: GO terms for D-curves cluster 2; Tab6: GO terms for D-curve cluster 4; Tab7: GO terms for U-curve cluster 1; Tab8: GO terms for U-curve cluster 2; Tab9: Membership of genes in D-curve; Tab10: Membership of genes in U-curve; Tab11: Differences of membership between two curves.

SI Dataset S2 (Supplementary_ASD_Brain.xlsx)

ASD Brain data results. Tab1: Differential network (DN) genes in 10 significant cell-types; Tab2: Leverage genes in 10 significant cell-types; Tab3: DN genes with marker genes and housekeeping genes for 4 neuron layers; Tab4: Leverage genes with with marker genes and housekeeping genes for 4 neuron layers.

References

1. H Dai, L Li, T Zeng, L Chen, Cell-specific network constructed by single-cell rna sequencing data. *Nucleic acids research* **47**, e62–e62 (2019).
2. J Tian, J Wang, K Roeder, Esco: single cell expression simulation incorporating gene co-expression. *Bioinformatics* **Epub ahead of print** (2021) btab116.
3. G Iacono, R Massoni-Badosa, H Heyn, Single-cell transcriptomics unveils gene regulatory network plasticity. *Genome Biol* **20**, 110 (2019).
4. M Ashburner, et al., Gene ontology: tool for the unification of biology. *Nat. genetics* **25**, 25–29 (2000).
5. F Supek, M Bošnjak, N Škunca, T Šmuc, Revigo summarizes and visualizes long lists of gene ontology terms. *PloS one* **6**, e21800 (2011).
6. D Velmeshev, et al., Single-cell genomics identifies cell type–specific molecular changes in autism. *Science* **364**, 685–689 (2019).
7. Y Baran, et al., Metacell: analysis of single-cell rna-seq data using k-nn graph partitions. *Genome biology* **20**, 1–19 (2019).
8. DS Matteson, NA James, A nonparametric approach for multiple change point analysis of multivariate data. *J. Am. Stat. Assoc.* **109**, 334–345 (2014).
9. S Ghazanfar, et al., Investigating higher-order interactions in single-cell data with schot. *Nat Methods* **17**, 799–806 (2020).
10. SS Huppert, M Iwafuchi-Doi, Molecular regulation of mammalian hepatic architecture. *Curr. topics developmental biology* **132**, 91–136 (2019).
11. LF Chu, et al., Single-cell rna-seq reveals novel regulators of human embryonic stem cell differentiation to definitive endoderm. *Genome biology* **17**, 173 (2016).
12. D Polioudakis, et al., A single-cell transcriptomic atlas of human neocortical development during mid-gestation. *Neuron* **103**, 785–801 (2019).
